# Quantifying the Spatial Distribution of Series Resistance in Monolithic Perovskite/Silicon Tandem Solar Cells Using Voltage‐Dependent Photoluminescence Imaging

**DOI:** 10.1002/smll.202513958

**Published:** 2026-02-27

**Authors:** Oliver Fischer, Anh Dinh Bui, Yan Zhu, Shuai Nie, Tanushree J. B. Nath, Yi Hui Hou, Wei Wang, Khoa Nguyen, Ary Anggara Wibowo, Jann B. Landgraf, Juliane Borchert, Florian Schindler, Heping Shen, Klaus Weber, Hieu T. Nguyen, Stefan W. Glunz, Ziv Hameiri, Daniel Macdonald, Martin C. Schubert

**Affiliations:** ^1^ Fraunhofer Institute for Solar Energy Systems ISE Freiburg Germany; ^2^ Chair for Photovoltaic Energy Conversion Department of Sustainable Systems Engineering INATECH University of Freiburg Freiburg Germany; ^3^ School of Engineering The Australian National University Canberra Australia; ^4^ School of Photovoltaic and Renewable Energy Engineering University of New South Wales Sydney Australia; ^5^ Cluster of Excellence *liv*MatS @ FIT – Freiburg Center for Interactive Materials and Bioinspired Technologies University of Freiburg Freiburg Germany

**Keywords:** advanced characterization, finite element simulation, perovskite/silicon tandem solar cell, photoluminescence imaging, series resistance imaging

## Abstract

To enhance the performance of monolithic perovskite/silicon tandem solar cells toward their theoretical limits and enable commercial‐scale deployment, it is essential to quantify local power losses and identify their physical origins. In this study, we apply a method to extract the local tandem series resistance (LTR_S_), a key contributor to the performance degradation of perovskite/silicon tandem devices. The method is based on bias‐voltage‐dependent photoluminescence (PL) imaging under two different illumination intensities, coupled with the generalized Planck's law. Finite element simulations demonstrate the robustness of the method under a range of realistic conditions, including current mismatch, low shunt resistance, and luminescence coupling effects. When exemplarily applied to a high‐efficiency perovskite/silicon tandem device with a power conversion efficiency *PCE* of 29%, the method reveals that approximately 1.9% absolute efficiency loss can be attributed to resistive effects. We further investigate the influence of the transient behavior of perovskites on LTR_S_ measurements using a metastable device. The results show that, even for unstable samples, reliable estimations of LTR_S_ can be achieved if an appropriate stabilization protocol is employed. These findings establish PL imaging as a powerful diagnostic tool for identifying performance‐limiting regions and guiding the design and processing improvements of next‐generation tandem photovoltaics.

## Graphical abstract

Spatially resolved series resistance in monolithic perovskite/silicon tandem solar cells is extracted using voltage‐dependent photoluminescence imaging. The method is robust against current mismatch, shunt resistance, and luminescence coupling. Even in a high‐performance 29% efficiency device, resistive losses contribute up to 1.9% absolute efficiency loss, highlighting the importance of diagnosing and minimizing series resistance for further performance improvements.

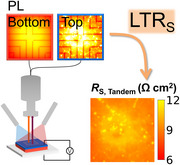

## 1 Introduction

Crystalline silicon solar cell technology is approaching its theoretical power conversion efficiency *PCE* limit of 29.4% [1], with the current champion device achieving *PCE* of 27.81% [2]. However, even at this theoretical limit, a silicon solar cell can harvest only about one‐third of the incident solar power. To accelerate the global transition toward net‐zero emissions and make the fabrication of photovoltaic modules more sustainable, it is essential to develop photovoltaic technologies that capture more of the solar spectrum. Monolithic perovskite/silicon tandem solar cells have emerged as a highly promising route. This device combines a low‐cost, solution‐processable perovskite top cell with a mature silicon bottom cell, exploiting existing silicon infrastructure while enhancing the overall efficiency. Recently, this tandem technology has achieved a certified *PCE* of 34.85% [2, 3], surpassing the theoretical limit of any single junction solar cell. Despite this great progress, current devices still fall short of their potential limit (37.8%–43.2%, depending on model assumptions) [4, 5, 6, 7], indicating substantial room for performance enhancement. Another major challenge is scalability. While most high‐efficiency tandem cells are fabricated on small areas (≤1 cm^2^), record crystalline silicon cells exceed 130 cm^2^ in active area [2]. Scaling up perovskite/silicon tandem devices often leads to performance losses – e.g., for identical structures and materials, increasing the active area from 1 cm^2^ to 16 cm^2^ reduced efficiency from 28.7% to 26.3% [8]. Furthermore, recent studies have reported annual degradation rates of up to 20% for state‐of‐the‐art tandems under outdoor conditions [9], far above the <1% required for commercial viability. These issues highlight the need for further optimization of both device architecture and material interfaces to boost efficiency and enhance long‐term stability. Achieving this goal requires a detailed understanding of the performance‐limiting factors and degradation mechanisms, which in turn demands advanced, sophisticated characterization methods [10].

The current–voltage (*j*−*V*) characteristic measured under standard 1 sun equivalent illumination remains the primary diagnostic tool for evaluating solar cell performance. Key parameters such as short‐circuit current density *j*_SC_, open‐circuit voltage *V*_OC_, fill factor *FF*, and *PCE* are extracted from it. Additional measurements such as *jV* curves under varying illumination intensity or *Suns*‐*V*_OC_ can be used to estimate series resistance *R*_S_ [11, 12, 13, 14]. However, these methods lack spatial resolution and cannot resolve local variations or process flaws. This is a major limitation for perovskite top cells, which are well‐known for their significant spatial inhomogeneity. Such inhomogeneities not only degrade performance, but can also accelerate long‐term degradation [15, 16, 17]. In addition, the spatial resolution is a desired piece of information for the root cause analysis of *R*_S_. While often several types of defects can influence global electric parameters in a similar way, the shape of a defect can point to the production step that has failed. Hence, measurement methods alternative to *jV* measurements are required to offer insight into spatially distributed effects that are critical for device optimization and reliability.

Photoluminescence (PL) imaging has become widely used as a non‐destructive and rapid characterization method in the field of silicon photovoltaics, both in laboratory research and industrial in‐line quality control [18, 19]. PL intensity contains rich information about device operation, enabling the extraction of key parameters such as the implied open‐circuit voltage i*V*_OC_, implied fill factor i*FF*, and ideality factor *n*_id_ [20, 21, 22, 23]. More recently, researchers have started applying PL imaging to analyze power loss and performance in perovskite/silicon tandem solar cells [24, 25, 26, 27]. However, current applications remain limited in scope, potentially due to several complicating factors that are unique to tandem architectures. First, the transient behavior of perovskite materials can lead to misinterpretation when extracting steady‐state parameters such as i*V*_OC_ [28]. Second, current mismatch between the subcells can distort the PL signal, complicating background correction and parameter analysis. Third, luminescence coupling between the subcells introduces additional uncertainty, as radiative recombination from the top cell may optically excite the bottom cell [29, 30]. These challenges highlight the need for careful interpretation of PL data in tandem devices and justify further efforts to develop robust PL‐based diagnostics tailored to these architectures. Independent of this work, Leon et al. [31] very recently developed a luminescence‐based method to image *R*_S_ of tandem solar cells. However, this approach requires the knowledge of *R*_S_ of the bottom solar cell and assumes a negligible influence of shunts.

In this work, we present a comprehensive approach formerly applied by Nesswetter et al. [32] to III‐V solar cells for perovskite/silicon tandem solar cells, combining finite‐element simulations and experiments to advance the use of PL imaging as a diagnostic tool for extracting one of the most performance‐critical parameters in monolithic tandem solar cells: the local series resistance *R*_S(*xy*)_. Since *R*_S_ significantly influences the *FF* and thus the overall *PCE*, it is crucial to minimize it for improving device performance. However, the origin of *R*_S_ is often complex, arising from multiple interdependent sources, such as emitter sheet resistance, contact resistance at the interface between the absorber and the electrode, and front‐side metallization [33, 34]. This complexity makes direct quantification and localization of *R*_S_ particularly challenging. By developing and validating a spatially resolved PL‐based method for perovskite/silicon tandem solar cells, we aim to provide a clearer picture of *R*_S_ distribution across the device area. Such insight is a critical step toward identifying dominant resistance pathways and enabling targeted design or process modifications. We begin by outlining the theoretical framework underlying the technique that was suggested by Nesswetter et al. [32] for the application to III‐V based multi‐junctions in the past. In a second step, we analyze the applicability of this method to perovskite/silicon tandem solar cells. This is necessary, considering that the structure, material composition, and material properties are different for perovskite/silicon tandem solar cells and III‐V tandem solar cells. Additionally, the work by Nesswetter et al. did not elaborate on the role of luminescence coupling and the role of luminescence emission from voltage‐independent carriers. However, both effects are known to occur in perovskite/silicon tandem solar cells. We use detailed simulations to examine how phenomena frequently observed in perovskite/silicon tandem solar cells affect the method's accuracy and validate the method. We find that the method is robust against the current mismatch, shunt resistance *R*_SH_, and luminescence coupling. Finally, we demonstrate the application of this method for the first time on perovskite/silicon tandems. The uncertainties associated with the unavoidable metastability of the perovskite subcell are investigated and minimized by a suitable measurement protocol.

## 2 Method Description

### 2.1 Single‐Junction Solar Cells

In any photovoltaic device, *R*_S(*xy*)_ can be calculated by dividing the difference between the local internal voltage *V*_INT(*xy*)_ and the terminal voltage *V*_EXT_ by the local current density *j*_(*xy*)_, as shown in Equation (1):

(1) $$R_{S\left(xy\right)}=\frac{V_{\mathrm{INT}\left(xy\right)}-V_{\mathrm{EXT}}\;}{j_{\left(xy\right)}}$$

*V*_INT(*xy*)_ corresponds to the local quasi‐Fermi level splitting *QFLS* divided by the elementary charge *q*. *j*_(*xy*)_ can be expressed as the difference between the generation current density *j*_GEN_ and the local dark current density *j*_DARK(*xy*)_; *j*_(*xy*)_ =  *j*_GEN_ − *j*_DARK(*xy*)_. *j*_GEN_ can either be approximated by *j*_SC_ or determined from the integrated product of the absorptance *a*(λ) and the spectrum of the illumination source *ϕ*_PH_(λ) Equation (2):

(2) $$j_{\mathrm{GEN}}=\int a\left(\lambda \right)\;s\phi_{\mathrm{PH}}\left(\lambda \right)\;d\lambda$$

Note that *R*_S(*xy*)_ defined by Equation (1) summarizes various resistive transport losses, e.g., from charge transport layers, transparent conductive oxides, and metal fingers, and is considered as a lumped resistance. Using the voltage and current differences between two operating conditions (changing the illumination intensity and the bias voltage) of the solar cell, we can modify Equation (1) and establish the following relationship:

(3) $$R_{S\left(xy\right)}=\frac{\Delta V_{\mathrm{INT}\left(xy\right)}-\Delta V_{\mathrm{EXT}\left(\mathrm{xy}\right)}\;}{\Delta j_{\mathrm{GEN}}-\;\Delta j_{\mathrm{DARK}\left(xy\right)\;}}$$

Following the approach from Kampwerth et al. [35], we simplify this equation, selecting two operating conditions, A and B of the solar cell, with different illumination intensities *I*_PH_ and different terminal bias voltages *V*_EXT,A_ and *V*_EXT(*xy*),B_ but same internal voltage, which results in Δ*V*_INT(*xy*)_ =  0. Condition A is typically pre‐selected under standard operating conditions (e.g., one sun equivalent illumination intensity), and the maximum power point voltage *V*_MPP_. Hence, we have *I*_PH,A_  =  1 suns and *V*_EXT,A_ = *V*_MPP_. The illumination intensity of condition B is usually chosen significantly different from condition A in order to improve the signal‐to‐noise ratio, but in the same order of magnitude to avoid non‐linear effects, e.g., *I*_PH,B_ = 0.5 suns. The optimal choice for *I*_PH,B_ might vary for different measurement systems and different tested solar cells. With this free selection of measurement parameters, *V*_EXT(xy),B_ is fixed and must be tuned to fulfil the desired condition *V*_INT(xy),A_ = *V*_INT(*xy*),B_. Note that this is equivalent to ensuring identical PL intensity for conditions A and B, and that it is always possible to find a suitable *V*_EXT(*xy*),B_ to fulfill this requirement. However, *V*_EXT(xy),B_ can vary for different positions (*x*_1_ *y*_1_) and (*x*_2_ *y*_2_) on the solar cell and is hence a local variable. Since Δ*V*_INT(*xy*)_  = 0 by construction, and the terminal voltage differs only slightly between conditions A and B, the effects of injection‐dependent series resistance described elsewhere [36, 37] are minimized, and we can assume that *R*_s(*xy*)_ remains effectively unchanged for the different conditions and *R*_S(*xy*),A_  =  *R*_S(*xy*),B_  = *R*_S(*xy*)_. Furthermore, Δ*V*_INT(*xy*)_  =  0 implies that Δ*j*_DARK(*xy*) _= 0, since identical internal voltages result in similar dark current densities. This allows us to simplify Equation (3) for single‐junction devices, which yields:

(4) $$R_{S\left(xy\right)}=\frac{-\Delta V_{\mathrm{EXT}\left(xy\right)}}{\Delta j_{\mathrm{GEN}}}=\frac{\;V_{\mathrm{EXT},A}-V_{\mathrm{EXT}\left(xy\right),B}\;}{j_{\mathrm{GEN},B}-j_{\mathrm{GEN},A}}\;$$

### 2.2 Dual‐Junction Solar Cells

For two‐terminal tandem solar cells, the situation is more complex since the internal voltage consists of contributions from both the top and bottom subcells. As Nesswetter et al. [32] show, this method is still applicable. For two‐terminal tandem solar cells, the internal voltage is expressed as:  *V*_INT(*xy*)_ =  *V*_INT(*xy*),TOP_ +  *V*_INT(*xy*),BOT_ with the local internal voltage of the top cell *V*_INT(*xy*),TOP_ and the local internal voltage of the bottom cell *V*_INT(*xy*),BOT_.

By substituting the relationship between the total internal voltage versus the subcell's internal voltages in Equation (3), we obtain Equation (5).

(5) $$R_{S\left(xy\right)}=\frac{\Delta V_{\mathrm{INT}\left(xy\right),\mathrm{TOP}}+\Delta V_{\mathrm{INT}\left(xy\right),\mathrm{BOT}}-\Delta V_{\mathrm{EXT}\left(xy\right)}\;}{\Delta j_{\mathrm{GEN}}-\;\Delta j_{\mathrm{DARK}\left(xy\right)\;}}$$

For tandem solar cells, condition B can be selected in a way that Δ *V*_INT(*xy*),TOP_ =  0 [1]. Then, also Δ *j*_DARK(*xy*),TOP_ =  0 holds as for the single junction solar cell. Noting that Δ*j*_GEN_ −  Δ*j*_DARK(*xy*)_  =  Δ*j*_GEN,TOP_ −  Δ*j*_DARK(*xy*),TOP_ for a monolithic two‐terminal tandem solar cell, we can adapt Equation (5) with the choice of condition B to [1]:

(6) $$R_{S\left(xy\right)}=\frac{\Delta V_{\mathrm{INT}\left(xy\right),\mathrm{BOT}}-\Delta V_{\mathrm{EXT}\left(xy\right)}\;}{\Delta j_{\mathrm{GEN},\mathrm{TOP}}}$$

As for the single‐junction solar cell, the approximation of Δ*j*_GEN,TOP_ by the difference in short‐circuit current Δ*j*_SC_ for the different illumination intensities is possible for the tandem solar cell. However, a top cell limitation is required during the measurement of *j*_SC_ and *j*_SC_ of the tandem deviates from the short‐circuit current of the top cell *j*_SC,TOP_ for tandem solar cells with a low parallel resistance in the top cell due to the reverse bias in the top cell under the current limiting condition. This can be resolved by performing spectrometric measurements [38, 39, 40] instead. The alternative determination of Δ*j*_GEN,TOP_ from the absorptance of the top cell *a*_TOP_(λ) and ϕ_PH_(λ) works analogous to the single‐junction solar cell with no further restrictions.

In contrast to Equation (4) for a single junction solar cell, the additional factor Δ*V*_INT(*xy*),BOT_ remains in Equation (6) and needs to be determined. For this, the relation between the internal voltage and the PL intensity $I_{\mathrm{PL}(xy)}=C\exp\left(\frac{q\;V_{\mathrm{INT}(xy)}}{k_{B}T}\right)$ can be employed with *C* being a calibration constant that depends on the absorptance of the absorber and comprises properties of the optical components of the measurement system, as well as physical constants. While it is possible to extract *V*_INT(xy),BOT_ by determining *C* [25], *C* may also remain unknown to determine Δ*V*_INT(*xy*),BOT_ as long as the absorptance of the sample at conditions A and B remains similar within the wavelength ranges of the light sources and of the PL detection range, and *C* does not change [41, 42, 43]. This has the advantage that Δ*V*_INT(*xy*),BOT_ can be determined independent of local optical interference effects due to, e.g., lateral thickness variations. With this, Δ*V*_INT,(xy), BOT_ can be determined by measuring *I*_PL(*xy*),BOT_ of the bottom cell under condition A and B and as Nesswetter et al. [32] outline, Equation (6) can be reformulated as:

(7) $$R_{S\left(xy\right)}=\frac{\frac{k_{B}T}{q}\;\ln\left(\frac{I_{\mathrm{PL}\left(xy\right),\mathrm{BOT},\;B}}{I_{\mathrm{PL}\left(xy\right),\mathrm{BOT},\;A}}\right)+V_{\mathrm{EXT},A}-V_{\mathrm{EXT}\left(xy\right),\;B}}{j_{\mathrm{GEN},\mathrm{TOP},B}-j_{\mathrm{GEN},\mathrm{TOP},A\;}}\;$$

We refer to the result of Equation (7) as the *local tandem R_S_* (LTR_S_).

It is worth noting that the derivation of the LTR_S_ method itself is independent of the current matching condition. This is based on the assumption that any layer between the subcells, such as the transparent conductive oxide recombination layer have very high sheet resistances, not allowing for significant lateral current flows within each subcell. If this assumption is not met, Δ*j*_GEN,TOP_ −  Δ*j*_DARK(*xy*),TOP_  =  Δ*j*_GEN,BOT_ −  Δ*j*_DARK(*xy*),BOT_ no longer holds, and the error of the extracted *R*_S(*xy*)_ increases. Another important remark is that the LTR_S_ method is independent of the investigated solar cell size. Although achieving homogeneous illumination becomes technically more challenging and a larger field of view of the camera compromises image resolution, the method can be applied to tandem solar cells without any fundamental size limitation.

### 2.3 Background in PL Images

Since the PL images acquired during this procedure are potentially taken while a significant portion of the generated current is extracted, voltage‐independent carriers can have a significant influence on the measured PL signal. With voltage‐independent carriers, we refer to carriers that are not extracted from the solar cell even under reverse bias conditions. These carriers may contribute to the luminescence signal if they recombine radiatively [44, 45]. To prevent measurement artefacts in the *R*_S(*xy*)_ determination, all PL images must be corrected by subtracting the background signals, including emissions from voltage‐independent carriers, as we discussed in our previous work for single‐junction perovskite solar cells [37]. While Nesswetter et al. did not investigate this effect in detail in their work, the most common practice for single‐junction solar cells is to record the PL image under the short‐circuit condition or a slight negative bias voltage, ensuring that all voltage‐dependent carriers are extracted. This allows for a pixel‐wise background correction. For tandem solar cells with current mismatch, a similar approach can be applied to the current‐limiting subcell. However, for the subcell with a higher generated current, a significant number of voltage‐dependent carriers may remain unextracted even at a negative terminal voltage, since this subcell typically operates under positive bias while the other subcell is limiting the overall current of the tandem. Therefore, capturing its background image under short‐circuit conditions of the tandem (0 V at the terminal) may lead to a substantial error. To address this, the illumination intensity of the other subcell needs to be increased until the investigated subcell is certainly the current‐limiting subcell in the device. For this, spectrometric measurements can be performed to determine the current matching point [38, 39, 40]. This enables proper carrier extraction under reverse bias and allows accurate background signal acquisition, also for tandem solar cells.

### 2.4 Measurement Algorithm

Based on the mathematical derivation above, the process of obtaining the *R*_S(*xy*)_ image for monolithic tandem solar cells using 1 and 0.5 sun equivalent illumination can be summarized in the following steps:

**1 Determination of the Generation Current**

Determine the generation current in the top cell Δ*j*_GEN,TOP_ for the two chosen illumination intensities, applying one of the three alternative options:

- Approximate Δ*j*_GEN,TOP_ from the difference in *j*_SC_ under the two chosen illumination intensities of the top cell, while the bottom cell illumination intensity must be sufficiently high to ensure a top cell limiting condition. Correct for the overestimation of *j*_GEN,TOP_ when approximating it with *j*_SC_ of the tandem if a low shunt resistance is present in the top cell.
- Otherwise, determine Δ*j*_GEN,TOP_ performing spectrometric measurements.
- Alternatively, determine Δ*j*_GEN,TOP_ from the absorptance of the top cell and the illumination intensity of the top cell.

**2 Image Acquisition at Key Operating Points**:

- Condition A: Capture PL images of each subcell under 1 sun equivalent illumination, with the terminal voltage set to *V*_MPP_. Correct PL images by subtracting the background signal.
- Condition B: Acquire PL images of each subcell under 0.5 suns equivalent illumination intensity for a series of, e.g., 10 terminal voltages with *V*_EXT,i_ =  *V*_MPP_ + *i* · d*V* for image *i* and d*V* being a small voltage. Correct PL images by subtracting the background signal.

**3 Interpolation of Top Cell PL Signal**:

Perform an interpolation [3] for the relation of PL signal versus terminal voltage using the captured PL images for condition B of the top cell to determine *V*_EXT(*xy*),B_ where the luminescence intensity at condition B is the same as for condition A for the top cell.

**4 Interpolation of Bottom Cell PL Signal**:

Similarly, perform an interpolation of the dependence of the bottom PL signal on the terminal voltage to find the PL signal intensity *I*_PL(xy),BOT,  B_ of the bottom cell that corresponds to *V*_EXT(*xy*),B _ determined in the previous step.

**5** ***R***_**S**_ **Calculation**:

Using the parameters identified for conditions A and B, calculate the *R*_s_ image based on Equation (7).

**FIGURE 1:**
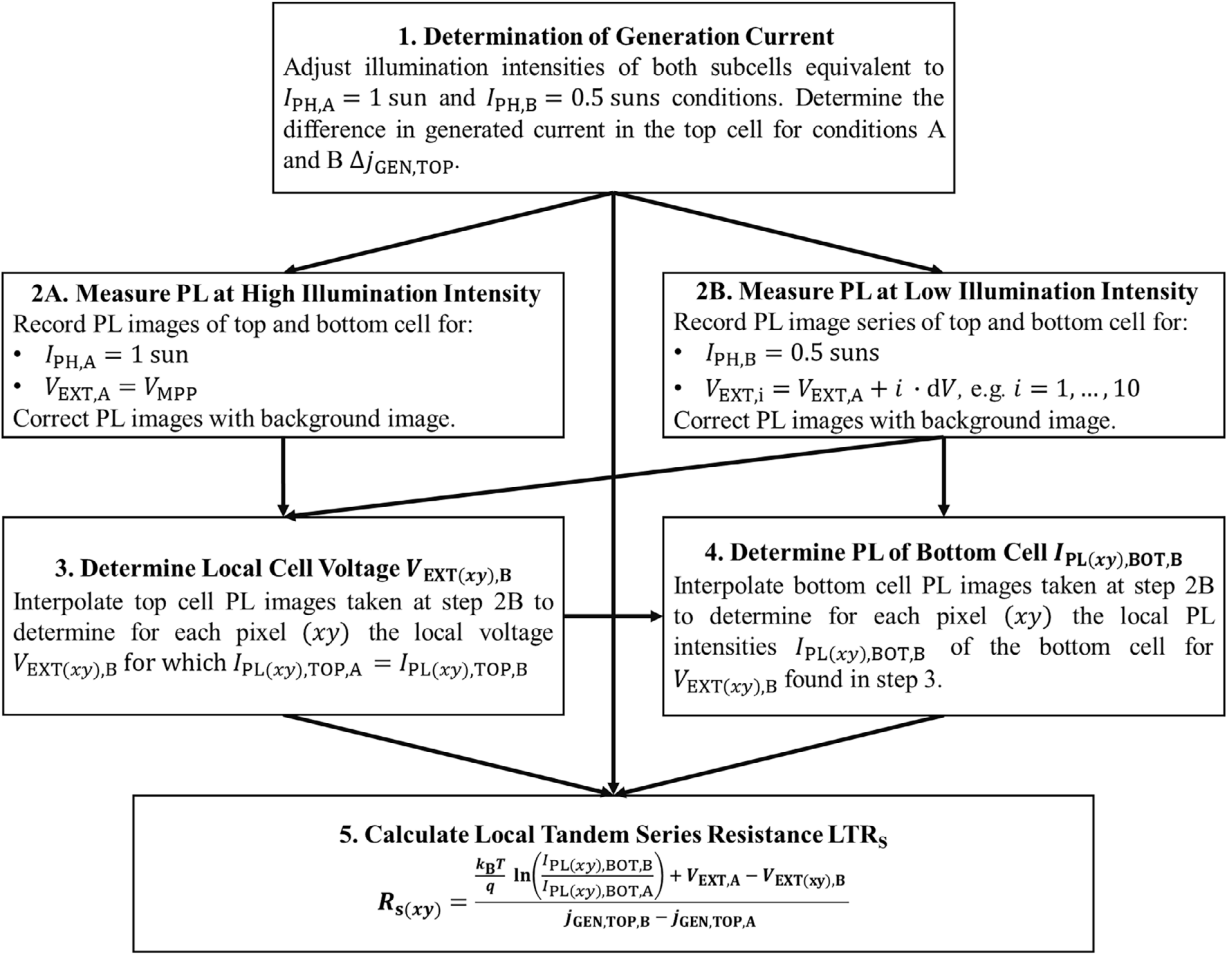
Workflow diagram illustrating the photoluminescence‐based method for quantifying the spatially resolved series resistance in monolithic tandem solar cells. The workflow describes the case where Δ *V*_INT(*xy*),TOP_ =  0 is chosen. The case for Δ *V*_INT(*xy*),BOT_ =  0 works analogously.

Figure 1 illustrates the complete procedure for quantifying the spatial distribution of series resistance in tandem solar cells.

Given the metastability of perovskite solar cells, it is prudent to implement precautions to ensure that the measured data accurately reflect *R*_S_ and are not affected by measurement artefacts. Considering the large variation in behavior among different perovskite compositions, no universal measurement protocol can be expected to apply in all cases. Nevertheless, the following precautionary measures help reduce hysteresis and aid in the detection of artefacts arising from the measurement process:

- The light sources should run continuously during the acquisition of PL images and must not be switched off between successive images.
- The bias voltage should be applied continuously, avoiding large voltage steps whenever possible.
- Sufficient stabilization time should follow each adjustment of the applied bias voltage.
- The duration of negative‐bias voltage application should be minimized. If device degradation seems likely, stabilization time may need to be reduced for this measurement step.
- Repeated PL measurements conducted under the same measurement conditions multiple times during the measurement routine may reveal device degradation and non‐stable measurement conditions.

In addition to these precautionary steps during the measurement procedure, measurements of the absorptance and the PL spectrum under continuous illumination are recommended to ensure the suitability of the tandem solar cell for the LTR_S_ measurement.

## 3 Results and Discussion

### 3.1 Simulation‐Based Method Demonstration and Evaluation

Perovskite/silicon tandem solar cells can not only suffer from transport losses, but also from low *R*_SH_. Additionally, different current matching conditions may be present depending on the spectral condition and the properties of the subcells. Luminescence coupling occurs in well‐performing solar cells at high internal voltages. It needs to be ensured that the results of the LTR_S_ method are independent of these effects. In this respect, we evaluate the outlined method through simulations using *Griddler 2.5 Pro* [46], a numerical simulation platform widely employed for spatially resolved analysis of solar cells. The use of simulation at this stage offers several key advantages: it provides direct access to subcell‐specific quantities, allows full control over critical parameters such as *j*_SC_, recombination properties, and parasitic resistances, and facilitates the introduction of spatial non‐uniformities in a controlled manner. These capabilities make *Griddler* an ideal environment for developing and validating new imaging‐based diagnostic techniques.

#### 3.1.1 Demonstration on an *R*_S_‐Feature‐Free Device

**FIGURE 2:**
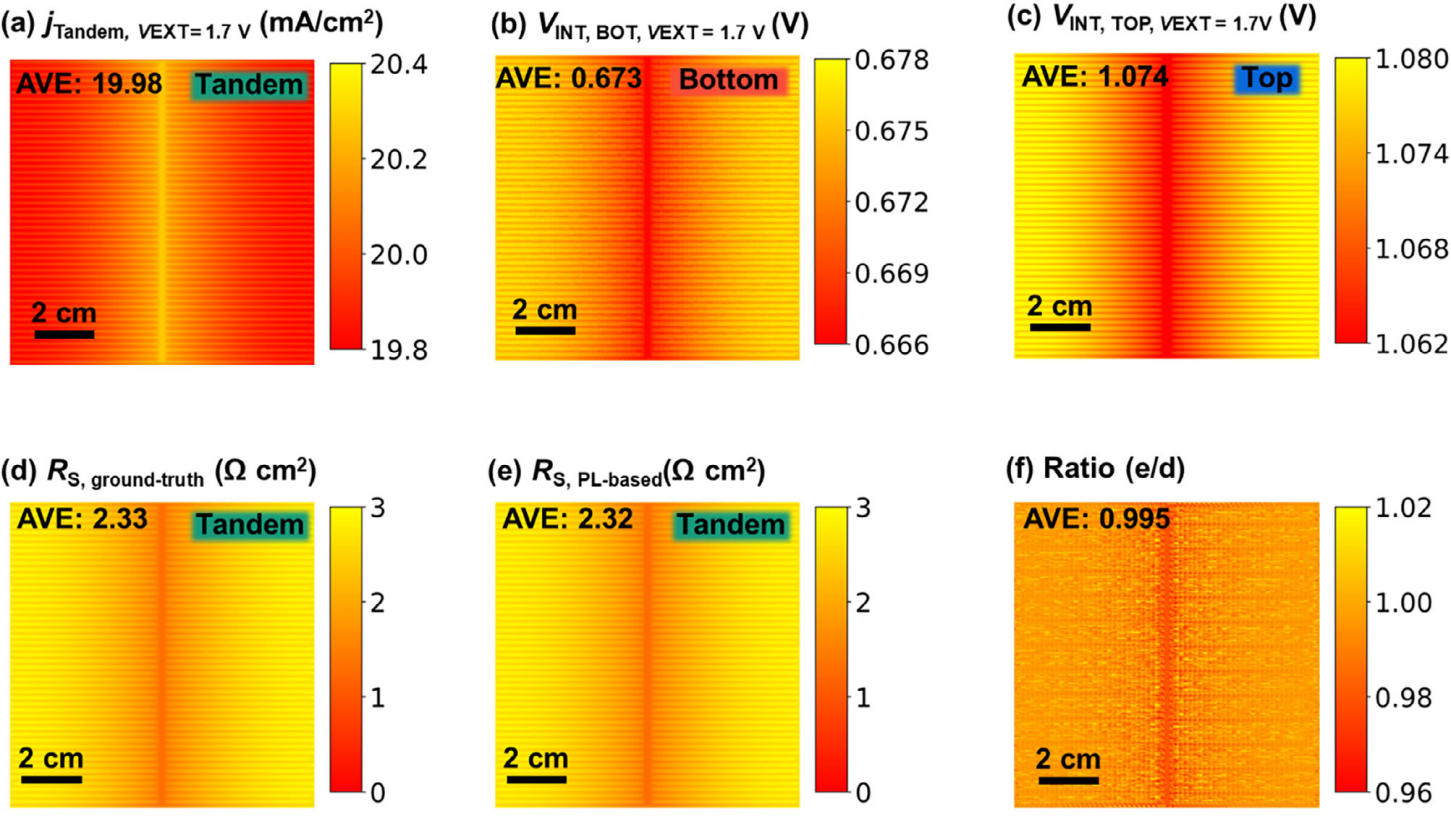
The device is simulated in current matching, and there is no intentionally introduced special feature. (a) Current density image of the tandem device, (b) internal voltage image at *V*_EXT_ =  1.7 V of the bottom cell, (c) internal voltage image at *V*_EXT_ =  1.7 V of top cell, (d) ground‐truth *R*_S_ image calculated from internal voltage images and current density images, (e) PL‐based *R*_S_ image, (f) ratio image between (d) and (e).

To start with the validation, we first demonstrate the method on a simulated tandem solar cell featuring high performance, current‐matched subcells, and no intentionally introduced spatial inhomogeneities in series resistance. Apart from the expected variation associated with the front contact grid geometry, the device exhibits a uniform. *R*_S_ distribution, providing a clean baseline for validating the accuracy and spatial resolution of the proposed method. Simulation parameters are detailed in Table S2. The *jV* characteristics are shown in Figure S1, yielding a *PCE* of 34%, which is close to the state‐of‐the‐art perovskite/silicon tandem solar cells [3, 47]. We take advantage of *Griddler*’s ability to access subcell‐specific quantities to validate the suggested method. Figure 2a–c present *j_xy_*, *V*_INT(*xy*),BOT_, and *V*_INT(*xy*),TOP_ of the simulated solar cell while the terminal voltage is set to 1.7 V and the illumination intensity is set to 1 sun. A local series resistance can be calculated using *Griddler*’s simulated data using Equation (8):

(8) $$R_{S\left(xy\right)}=\frac{V_{\mathrm{INT}\left(xy\right),\;\mathrm{BOT}}+V_{\mathrm{INT}\left(xy\right),\mathrm{TOP}}\;-\;V_{\mathrm{EXT}}}{j_{\mathrm{TANDEM}\left(xy\right)}}\;$$

The resulting simulated *R*_S_ image is presented in Figure 2d (hereafter referred to as ground‐truth *R*_S_). Near the central busbar, *R*_S_ is slightly lower than in surrounding regions – a physically intuitive result due to the shorter lateral transport path. The arithmetic mean of the simulated local *R*_S_ is 2.33 Ω cm^2^. However, *Griddler* also allows for determining the local PL intensities. This allows us to determine the local *R*_S_ image in Figure 2e that is calculated using the LTR_S_ method explained above (hereafter referred to as PL‐based *R*_S_). Note that since PL intensities from *Griddler* are based on the internal voltage that *Griddler* can access, also below fingers and busbars, a virtual PL signal is also available for areas that are covered with fingers and busbars. The arithmetic mean *R*_S_ value is 2.32 Ω cm^2^ for the PL based LTR_S_ calculations and agrees well with the reference value. The spatial variation shows negligible variation between the two *R*_S_ images as the ratio image in Figure 2f shows.

#### 3.1.2 Effect of Intentionally Introduced Feature *R*_S_

After demonstrating the method on an ideal device exhibiting only grid‐induced *R*_S_ variation, we move one step forward toward real‐world tandem solar cells, which often suffer from spatial heterogeneities in photovoltaic parameters, including *R*_S_ [48, 49, 50, 51]. To evaluate the sensitivity and accuracy of the method under more realistic conditions, we perform simulations on a modified device in which spatially localized *R*_S_ features were intentionally introduced in this section. The same simulation framework was used as in Section 3.1.1, with the only modification being the introduction of two distinct high‐*R*_S_ regions: feature R in the top cell (1.155 Ω cm^2^ higher than surrounding area) and feature s (0.315 Ω cm^2^ higher than the surrounding area) in the bottom cell. Importantly, the average *R*_S_ across the device was kept constant to ensure comparable *jV* performance (see Figure S2, Supporting Information).

**FIGURE 3:**
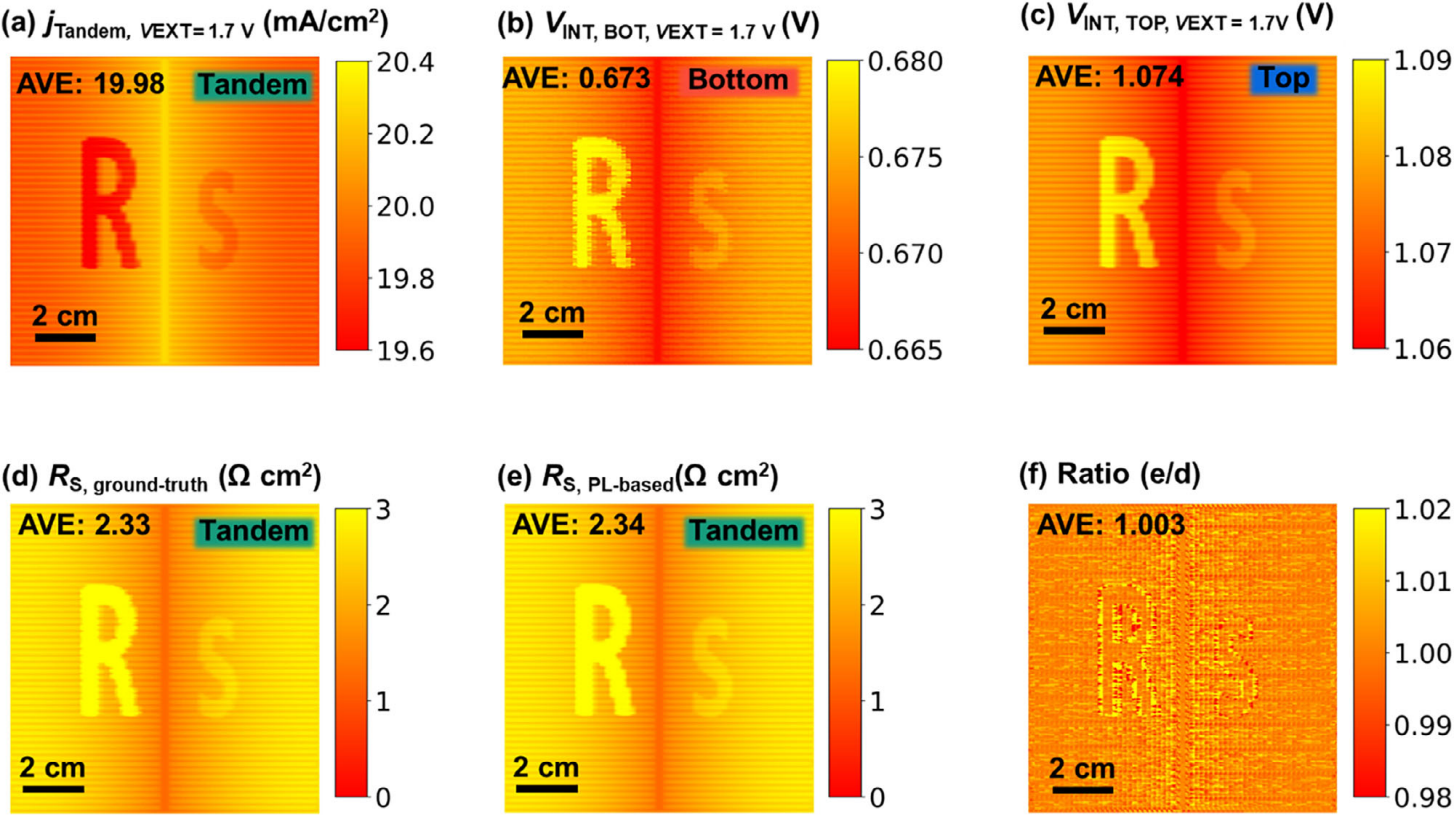
The device is simulated in current matching, and there are intentionally introduced special *R*_S_ features. (a) Current density image of the tandem device, (b) internal voltage image at *V*_EXT_ =  1.7 V of the bottom cell, (c) internal voltage image at *V*_EXT_ =  1.7 V of top cell, (d) ground‐truth *R*_S_ image calculated from internal voltage images and current density images, (e) PL‐based *R*_S_ image, (f) ratio image between (e) and (d).

As before, we simulate the local *j_xy_* (Figure 3a), the local *V*_INT(*xy*),BOT_ (Figure 3b), and the local *V*_INT(*xy*),TOP_ (Figure 3c) under standard conditions of 1 sun illumination and a terminal voltage of 1.7 V. The ground‐truth *R*_S_ image, calculated directly from internal voltages using Equation (8), is shown in Figure 3d. The PL‐based *R*_S_ image, obtained using the LTRS method, is shown in Figure 3e.

Since *R*_S_ impedes current flow regardless of which subcell it originates from, it significantly affects both the local current density and internal voltage distributions (i.e., PL intensity) under applied bias conditions. Consequently, both feature R in the top cell and feature s in the bottom cell are clearly resolved in both the simulated ground‐truth *R*_S_ image and the PL‐based *R*_S_ image. The spatial patterns and magnitudes show strong agreement between the two, as further supported by the ratio image in Figure 3f. These results confirm that the method retains its accuracy in the presence of moderate spatial inhomogeneities and can reliably identify and quantify *R*_S_ features originating from either subcell.

To further evaluate the robustness and spatial accuracy of our method, we exploit one of the key advantages of *Griddler*: the ability to precisely define and control internal series resistance values within individual subcells. As the primary modification between the setups in Sections 3.1.1 and 3.1.2 is the spatial distribution of internal *R*_S_, we specifically assess the method's sensitivity by comparing the difference in calculated *R*_S_ values between these two cases.

**FIGURE 4:**
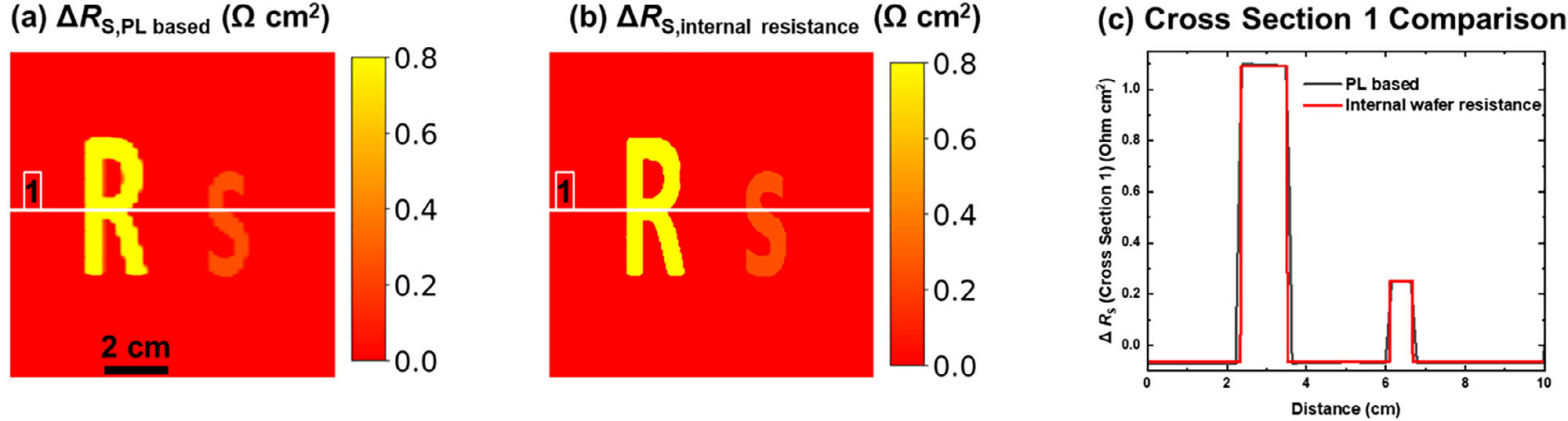
The device is simulated in current matching, and there are intentionally introduced special *R*_S_ features. (a) Δ*R*_S_ derived from PL‐based measurements, obtained by subtracting the *R_S_* image of a homogeneous device (Figure 2e) from that of a device with intentionally introduced *R*_S_ features (Figure 3e). (b) Corresponding Δ*R*_S_ map calculated from set up of internal wafer resistance (Equation (9)), serving as the ground‐truth reference (see Figure S3). (c) Line profile along cross Section 1 (indicated in a–b) comparing PL‐based and simulation‐derived Δ*R*_S_ values.

Using the PL‐based *R*_S_ image from Figure 2e (feature‐free case) and Figure 3e (feature‐introduced case), we compute a differential resistance image. This difference is presented in Figure 4a.

To benchmark this result, we also calculate the expected difference in local internal wafer resistance *R*_S,wafer_ based on the simulation inputs. This theoretical difference is defined through Equation (9) as:

(9) $$\begin{array}{c} \Delta R_{S,\exp\mathrm{ected}}=\left(R_{S,\mathrm{wafer},\mathrm{TOP},\mathrm{feature}}+R_{S,\mathrm{wafer},\mathrm{BOT},\mathrm{feature}}\right)-\\ \left(R_{S,\mathrm{wafer},\mathrm{TOP},\mathrm{no}\;\mathrm{feature}}+R_{S,\mathrm{wafer},\mathrm{BOT},\mathrm{no}\;\mathrm{feature}}\right) \end{array}$$

The corresponding simulated input images for internal *R*_S,wafer_ in the top and bottom subcells are shown in Figures S3a and S3b (Supporting Information). Figure 4b presents the resulting Δ*R*_S,expected _image.

To facilitate a direct comparison, a cross‐sectional profile is extracted from both Δ*R*_S,PL‐based_ and Δ*R*_S,expected _along the horizontal line marked in Figure 4a,b. As shown in Figure 4c, the PL‐derived Δ*R_S_*‐profile closely matches the expected values across the cross‐section. Minor deviations were observed in the measured differential *R*_S_ are primarily attributed to current redistribution effects dictated by the cell geometry and lateral transport pathways. Aside from these edge effects, the method shows high accuracy in capturing the spatial *R*_S_ changes introduced deliberately into either subcell. The baseline Δ*R*_S _in Figure 4c is negative since the background *R*_S_ was lowered with the introduction of the *R*_S_ features to keep overall average *R*_S_ and performance of the solar cell similar.

#### 3.1.3 Effect of Current Mismatch

Previously, we demonstrated the accuracy of the method under ideal current‐matched conditions. However, achieving perfect current matching in monolithic tandem devices is technically challenging in practice due to inevitable spectral and material variations. Therefore, it is critical to assess the robustness of the method under current‐mismatched conditions.

**FIGURE 5:**
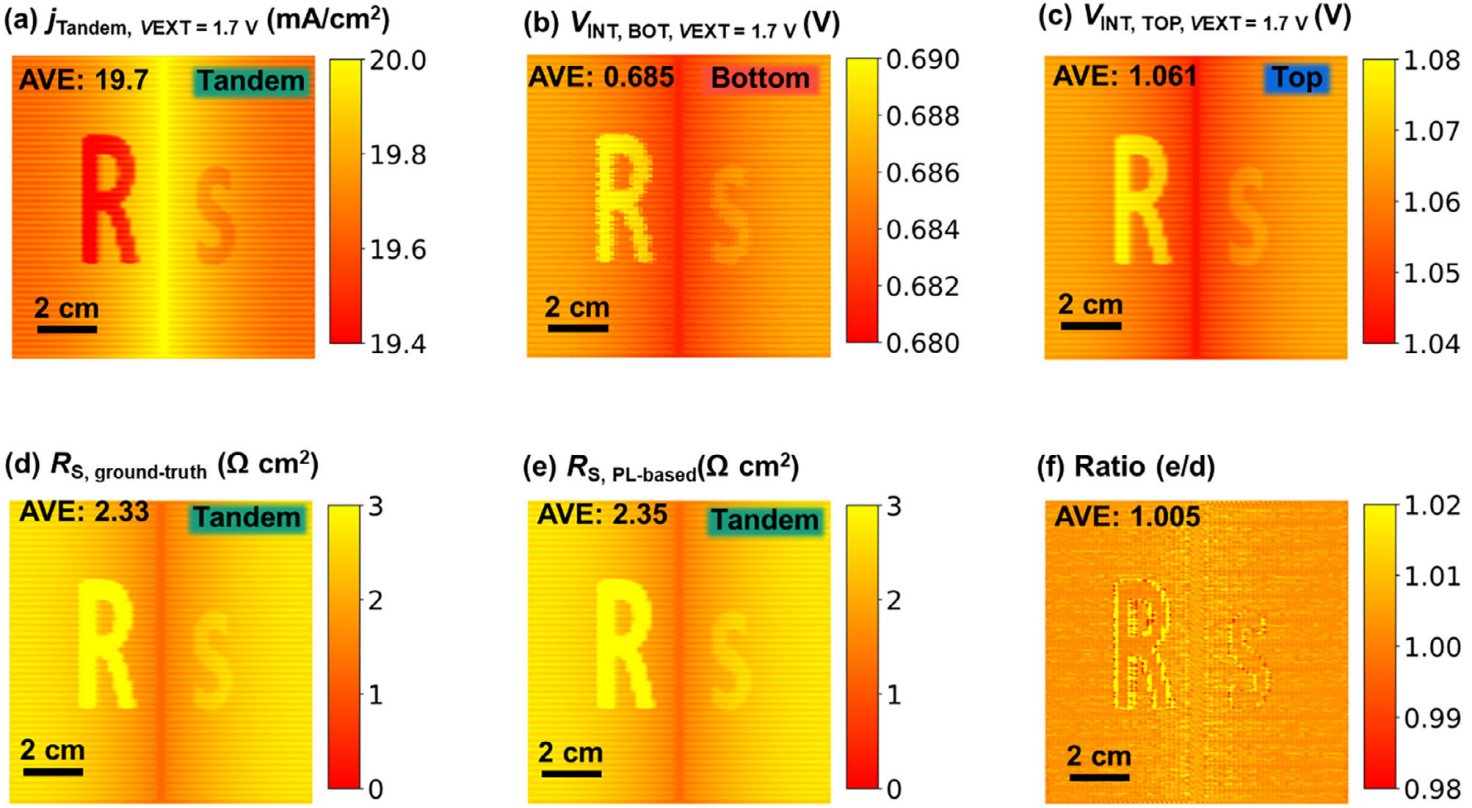
The device is simulated under a top cell limiting condition and with an intentionally introduced R_S_ feature. (a) Current density image of the tandem device, (b) internal voltage image at *V*_EXT_ =  1.7 V of the bottom cell, (c) internal voltage image at *V*_EXT_ =  1.7 V of top cell, (d) ground‐truth *R*_S_ image calculated from internal voltage images and current density images, (e) PL‐based *R*_S_ image, (f) ratio image between (e) and (d).

In this section, we evaluate the method on a tandem device with laterally non‐uniform *R*_S_ and current mismatch, where *j*_SC,TOP_  =  20.7 mA/cm^2^ and *j*_SC,BOT_  =  21.7 mA/cm^2^ are set for a top cell limiting condition. Simulation parameters are provided in Table S2, and the *jV* curve is shown in Figure S4. As in the previous section, we take advantage of *Griddler*’s ability to access subcell‐specific quantities and compute *j_xy_*, *V*_INT(*xy*),BOT_, and *V*_INT(*xy*),TOP_ of the top cell while the terminal voltage is set to 1.7 V and the illumination intensity is set to a spectrum similar to 1 sun that generates a current mismatch. The resulting images are shown in Figure 5a–c, respectively. Based on these data, the local ground‐truth *R*_S(*xy*)_ is calculated using Equation (8) as in the previous section. The spatial variations are deliberately introduced as distinguishing features in each subcell. The derived tandem *R*_S_ image is shown in Figure 5d. Notably, the key spatial features from both top and bottom cells are combined in the final *R_S_* image. This shows that the final *R*_S_ image reflects contributions from all layers, independent of the limiting subcell. The mean *R*_S_ of the tandem device is 2.33 Ω cm^2^. In comparison, we again use the luminescence intensity images of the subcells extracted from *Griddler* to calculate the *R*_S(*xy*)_ image according to the LTR_S_ method. The resulting image is shown in Figure 5e. Only minor deviations of below 1% that can probably be attributed to numerical errors are present, as the ratio image in Figure 5f shows. This indicates that the method can accurately resolve spatially varying series resistance, even under current mismatch conditions.

To further evaluate our method under varying mismatch conditions, we simulate two additional cases: (i) A bottom cell–a limiting case where the generation currents of top and bottom cells are 21.7 mA/cm^2^ and 20.7 mA/cm^2^, respectively. For this case, condition B was chosen in a way that the bottom cell PL signal was the same as for condition A, implying that the bottom cell generation currents and the PL images of the top cell were used in Equation (7). (ii) A strong top cell–limiting case, where the top cell generates only one‐third of the current of the bottom cell. For this case, condition B was again chosen to keep the top cell PL signal constant. The results, shown in Figures S5 and S6, respectively, reveal that the method consistently reproduces the ground‐truth *R*_S_ images with high fidelity, showing a deviation of below 1%. These findings confirm the robustness of the PL‐based approach, even under significant subcell mismatch, and highlight its suitability for diagnosing resistive losses in real‐world tandem architectures.

#### 3.1.4 Effect of Background Image Subtraction

**FIGURE 6:**
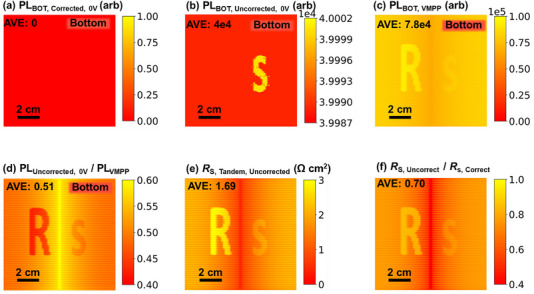
PL images of the bottom cell at (a) 0 V, the top cell is illuminated at 1.1 sun to make sure that the bottom cell is the limiting cell. (b) 0 V, top cell is illuminated at 1.0 sun (top cell is the limiting cell). (c) Bottom cell at *V*_MPP_ (top cell is limiting cell). (d) Ratio image between (b) and (c). (e) *R*_S_ image was calculated by using (b) as a dark correction. (f) Ratio image between the uncorrected and corrected *R*_S_ image from Figure 5c.

The current mismatch investigated in the previous section must be accounted for in the background image subtraction procedure. The importance of capturing the background image under the correct subcell limitation is investigated in this section. In our simulation, the top cell (initially the limiting cell) was set to a current density of 20.7 mA/cm^2^, while the bottom cell had 21.7 mA/cm^2^. To correctly capture the background image of the bottom cell under condition A, we increased the illumination intensity of the top cell to the equivalent of 1.1 suns, thereby making the bottom cell current‐limiting. Figure 6a shows the PL image of the bottom cell under the adjusted spectral condition for a terminal voltage of 0 V. Note that in the *Griddler* simulation, there are no voltage‐independent carriers; therefore, the PL signal under short‐circuit conditions is expected to be zero. However, as shown in Figure 6b, when reverse bias is applied without adjusting the illumination, the bottom cell still exhibits a substantial PL signal, with a mean value of 4.0 × 10^4^ counts, even though no voltage‐independent carriers are present. For comparison, the mean PL intensity of the bottom cell under *V*_MPP_ is only 7.8 × 10^4^ counts (Figure 6c). The ratio of non‐adjusted background PL to PL at *V*_MPP_ is shown in Figure 6d, with an average value of 0.51. The *R*_S_ image calculated using this uncorrected background is shown in Figure 6e, with a mean value of 1.69 Ω cm^2^. This represents a 30 % deviation from the expected *R*_S_ value, as illustrated in Figure 6f. It is important to emphasize that this simulation represents an idealized scenario, where parasitic emissions and voltage‐independent carrier effects are negligible. In real devices, these effects can be more pronounced, and the error introduced by an inaccurate background image could be even greater.

#### 3.1.5 Effect of Shunt Resistance

**FIGURE 7:**
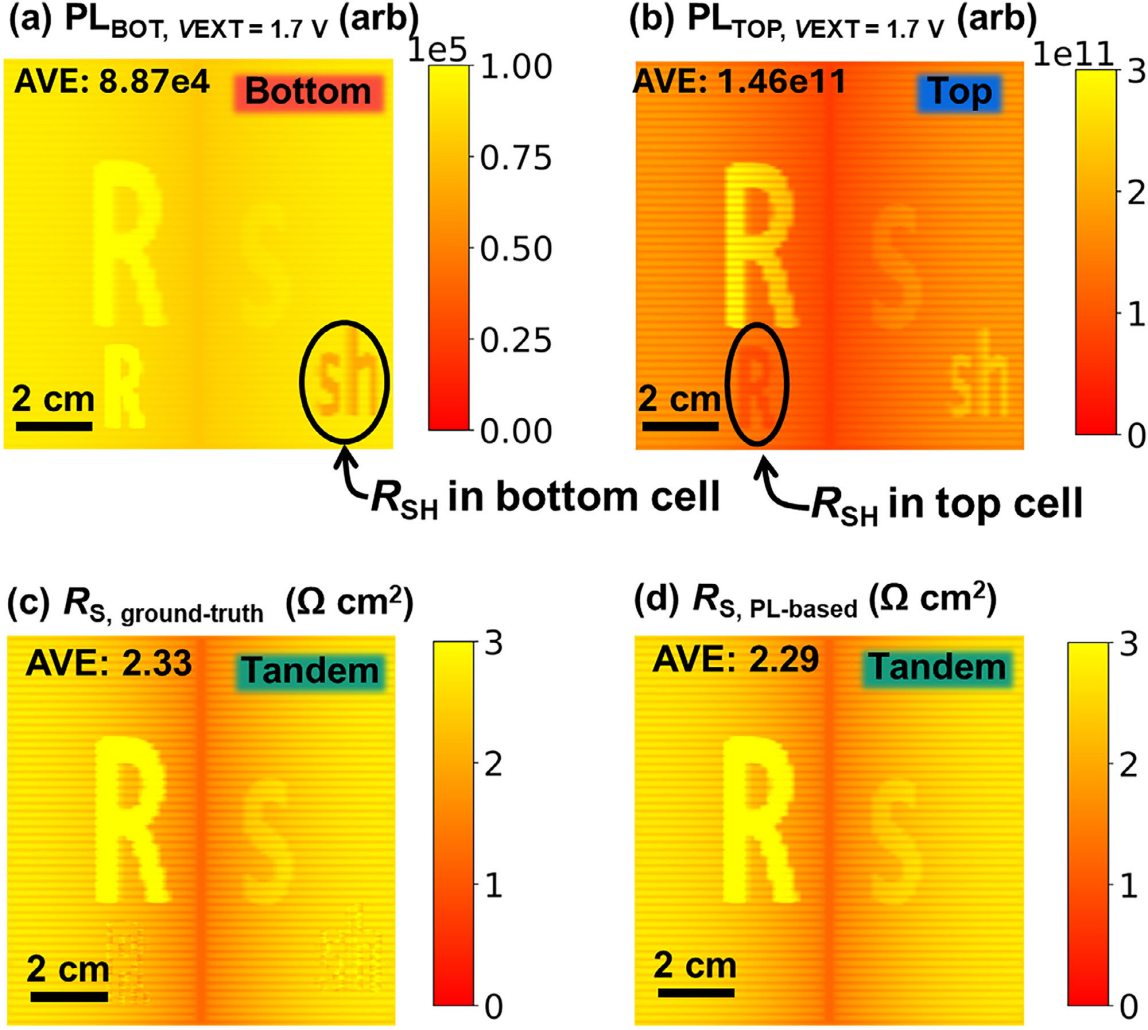
The device is simulated with an intentionally introduced *R*_SH_ feature. (a) PL image of the bottom cell at *V*_EXT_ =  1.7 V, (b) PL image of the top cell at *V*_EXT_ =  1.7 V, (c) internal voltage image at *V*_EXT _ =  1.7 V of top cell, (d) ground‐truth *R*_S_ image, (e) PL‐based *R*_S_ image.

Low *R*_SH_ can significantly reduce the *V*_OC_, and since PL intensity is exponentially dependent on *V*_OC_, even moderate variations in *R*_SH_ can lead to substantial spatial contrast in the PL image. Hence, the influence of *R*_SH_ on the LTR_S_ method needs to be investigated. Artificial shunt features were introduced into both top and bottom cells, with the corresponding *jV* curve shown in Figure S7a. Under open‐circuit conditions, the PL intensity is significantly affected by *R*_SH_, whereas the impact of *R*_S_ is negligible due to the absence of current flow across *R*_S_, as displayed in Figure S7b,c. Moreover, *R*_SH_ alters the current‐matching behavior between subcells, as also noted by Boccard et al. [52]. Under MPP bias, both *R*_S_ and *R*_SH_ manifest distinctly in PL images (Figure 7a,b). While *R*_S_ impedes carrier extraction and leads to a higher PL intensity under current extraction, *R*_SH_ provides alternative current paths and decreases *QFLS*, reducing PL intensity. Interestingly, interaction between subcells is evident: features of the top cell appear in the bottom cell's PL image and vice versa. The ground‐truth *R*_S_ image computed using Equation (8) and *Griddler*’s simulated subcell voltage/current data is shown in Figure 7c, while the corresponding *j*, *V*_INT,BOT_, and *V*_INT,TOP_ images are presented in Figure S7d–f, respectively. The PL‐based *R*_S_ image, derived from subcell luminescence data, is shown in Figure 7d. Despite the presence of *R*_SH_‐induced PL contrast, the PL‐derived *R*_S_ image remains in good agreement with the ground‐truth simulation, exhibiting only minor deviations of below 2% attributable to numerical noise. Importantly, while the ground‐truth *R*_S_ image (Figure 7c) shows minor spurious features near shunted regions, likely due to small uncertainties in converting PL to internal voltage, the PL‐based *R*_S_ image (Figure 7d) is largely unaffected by *R*_SH_. This highlights a key advantage of our approach: the method is selectively sensitive to *R*_S_ rather than *R*_SH_, enhancing its reliability in characterizing series resistance even in the presence of shunting.

#### 3.1.6 Effect of Luminescence Coupling

**FIGURE 8:**
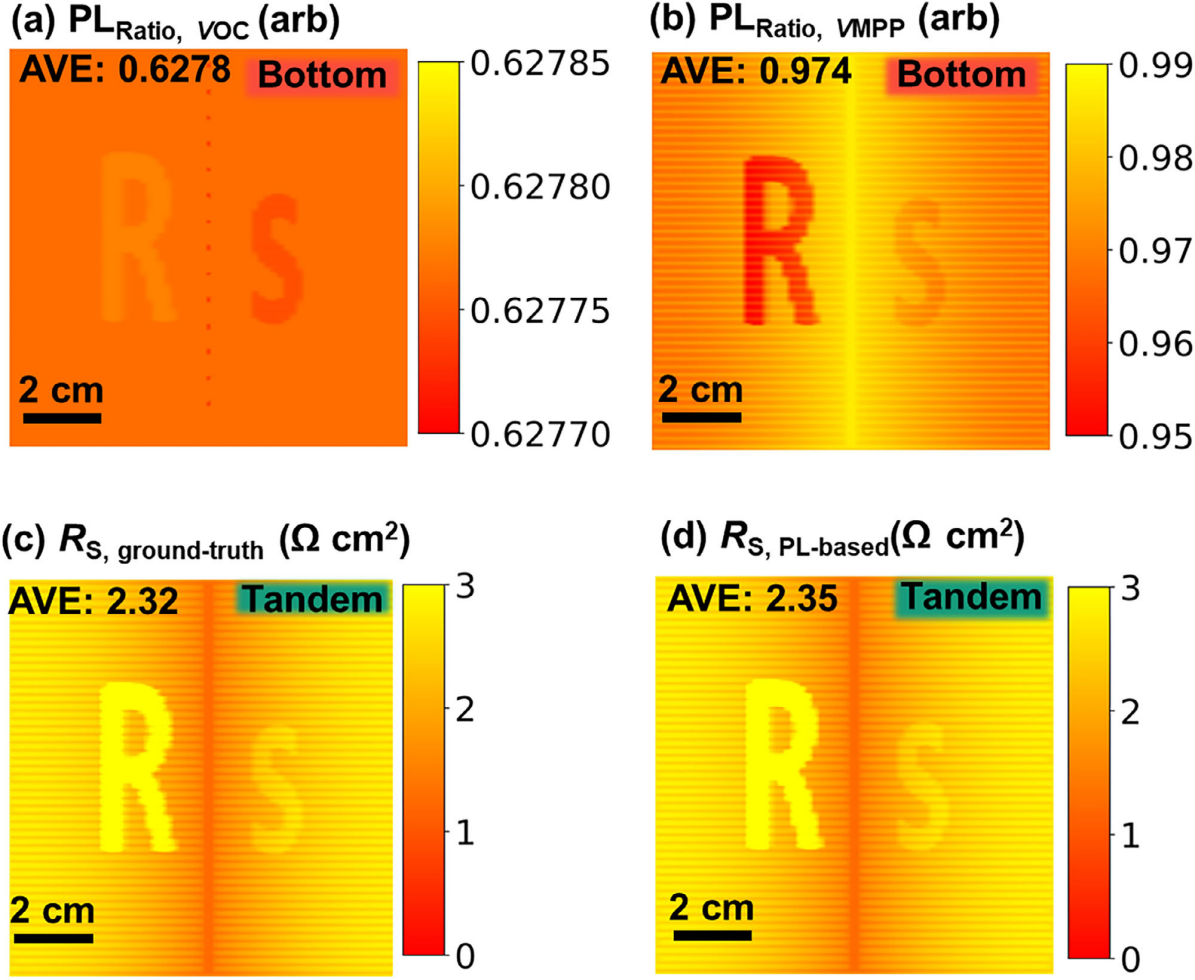
The device is simulated in the top cell, limititng with LC effect. (a) Ratio image between PL at *V*_OC_ of the bottom cell with and without the LC effect, (b) ratio image between PL at *V*_MPP_ of the bottom cell without and with LC effect, (c) ground‐truth *R*_S_ image, (d) PL‐based *R*_S_ image.

Luminescence coupling (LC) occurs when radiative recombination in the high‐bandgap top subcell emits photons that are subsequently reabsorbed by the low‐bandgap bottom subcell [30, 53]. This mechanism was shown to enhance the bottom‐cell current and potentially boost the energy yield of tandem devices, as demonstrated in literature [54]. Hence, in the last section of validating simulations, we evaluate the influence of LC on the method by enabling the LC feature in *Griddler* and setting an LC ratio (backside PL emission over total PL emission) of 87%, similar to typical values in literature [7, 30]. Additionally, we also adjust the simulation parameters in order to match approximately the radiative limit of the device, while keeping the tandem solar cell in a top cell‐limited condition and considering Auger recombination in the bottom cell. The simulation parameter details are tabulated in Table S2. The resulting *jV* curves of the device with and without LC effect are shown in Figure S8a and exhibit a slight increase in *V*_OC_, resulting in a marginal *PCE* improvement. To quantify the impact on PL response, we calculated the ratio of PL images without and with LC for the bottom cell at both *V*_OC_ and *V*_MPP_ conditions (Figure 8a,b). At *V*_OC_, where the radiative recombination rate of the top cell is the highest, PL intensity increases by 33.7% due to LC. At *V*_MPP_, this effect diminishes to 2.6%, aligning with the *jV* data.

To determine whether LC influences the *R*_S_ extraction, we computed the ground‐truth *R*_S_ image using simulated current density and internal voltage images (Figure S8b–d). The resulting *R*_S_ image (Figure 8c) shows negligible deviation from the LC‐free case with minor deviations below 1%, confirming that series resistance is unaffected by LC itself. Similarly, the *R*_S_ image extracted from PL images (Figure 8d) closely matches the ground‐truth image, aside from minor numerical discrepancies. These results demonstrate that, although LC slightly alters PL intensity, it does not affect the accuracy of *R*_S_ determination using our method.

To further probe the sensitivity of the method under more LC‐prone conditions, we simulated a second scenario where the bottom cell is current‐limiting—thus more likely to benefit from additional photon absorption via LC. The results (Figure S9) again show excellent agreement between PL‐derived and ground‐truth *R*_S_ images, reinforcing that the method remains robust regardless of the limiting subcell or LC strength. Since luminescence coupling influences the generated current of the bottom solar cell, condition A and B are chosen in this example in a way that the PL signal of the bottom solar cell is identical for the data shown in Figure S9, implying that the generated bottom cell current must be used for the denominator in Equation (7), to investigate the most critical case. Even under these conditions, although LC modifies the PL intensity, it does not impair the accuracy of the extracted *R*_S_ values when the generated current is correctly accounted for. It is worth noting, however, that the total generated current for the bottom cell includes both direct photogeneration and LC contributions in this simulation. While this is easily quantified in simulation environments such as *Griddler*, it presents challenges in experiments, as LC‐generated current depends on the top cell's bias. If the LC contribution is not included in the denominator of Equation (7) and *j*_GEN,BOT_ instead of *j*_GEN,TOP_ is used, it can result in an underestimation of *R*_S_. In our simulations, this would cause an error of approximately 20%.

To avoid this, a simple and practical solution is to adjust illumination conditions such that the top cell PL signal remains constant between conditions A and B. This ensures that the top cell's generated current—unaffected by LC—can be used as the reference in Equation (7), thereby eliminating any LC‐induced error in the extracted *R*_S_ image. Note that Δ*V*_INT(xy),BOT_ may be influenced by LC. However, this is correctly accounted for by measuring *I*_PL(*xy*),BOT,  A_ and *I*_PL(*xy*),BOT,  B_.

In summary, we systematically introduced current mismatch, shunt resistance, and luminescence coupling—three common non‐idealities in perovskite/silicon tandem solar cells—to demonstrate the robustness of the LTR_S_ method under realistic device conditions. Across all scenarios, the PL‐derived series resistance images closely matched the ground‐truth simulation values. This comprehensive validation highlights the method's applicability for diagnosing resistive losses in complex, real‐world tandem architectures that are investigated in the following.

### 3.2 Experimental Application on Perovskite/Silicon Tandem Solar Cells

Next, we apply the proposed method to quantify the spatial distribution of *R*_S_ in monolithic perovskite/silicon tandem solar cells with a structure displayed in Figure S10. Detailed information on the device fabrication is provided in Section Solar Cell Fabrication in the Supporting Information.

#### 3.2.1 Stable Tandem Device with Spatial Inhomogeneity

**FIGURE 9:**
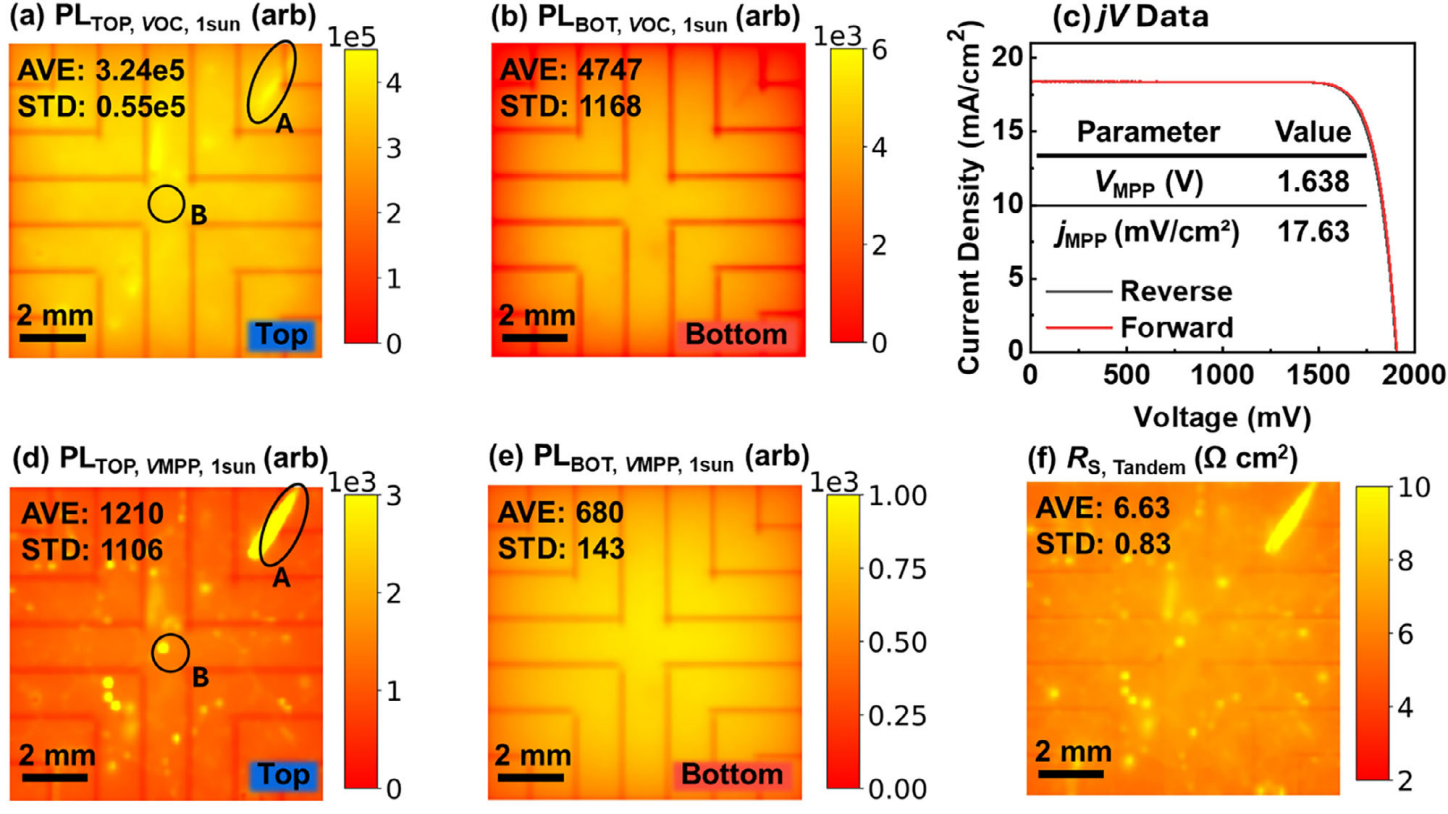
PL images at *V*_OC_ of (a) top cell, (b) bottom cell under 1 sun illumination. (c) Current voltage characteristic curve of the tandem device. PL images at *V*_MPP_ of (d) top cell, (e) bottom cell under 1 sun illumination. (f) *R*_S_ image of the device.

We begin with a high‐performance tandem device exhibiting a *PCE* of 29%, as shown by the *jV* curve in Figure 9c. The external quantum efficiency (EQE) of a similar device, presented in Figure S11a (Supporting Information), provides a relative EQE for each subcell. The *jV* parameters can be found in Table S3. Figure 9a,b,d,e displays PL images of the top and bottom subcells under *V*_OC_ and *V*_MPP_ conditions. Photoluminescence quantum yield of less than 0.5% is found for similar devices. Despite the good *PCE* and relatively uniform bottom‐cell behavior, the top cell exhibits a pronounced spatial heterogeneity. Several distinctive features are observed across the cell surface under *V*_MPP_ condition. For example, Feature A and Feature B, which are highlighted in Figure 9d display very high PL intensity under maximum power point conditions compared to the surrounding regions. In contrast, Feature A shows only slightly enhanced PL signal under *V*_OC_ conditions in Figure 9a (suggesting the presence of less non‐radiative recombination in this region), and Feature B, located near the center of the device, is not visible under *V*_OC_ conditions at all. This suggests that in these regions, charge carrier extraction efficiency is reduced due to some local defect. The comet tail shape of Feature A suggests that the defect in Feature A was caused by a spin coating process. A dust particle hindering the homogeneous spread of the solution could cause a hole in, e.g., the spin‐coated hole transport layer. The point‐shaped defect of Feature B could originate from agglomerates that formed in, e.g., the solution of the passivation agent applied to this solar cell, hindering charge carrier transport locally. To quantify the impact of these defects, we computed the spatial *R*_S_ distribution shown in Figure 9f. In line with the findings on PL‐intensity, the series resistance in Figure 9f is locally increased in the regions of Feature A and Feature B. The device exhibits a mean *R*_S_ of 6.63 Ω cm^2^ with a relative standard deviation of 12.5%. The dominant contribution to the spatial variation in *R*_S_ originates from the perovskite top subcell, as confirmed by correlating local *R*_S_ values with the local change in internal voltage with respect to variations in applied voltage (d*V*_int_/d*V*_ext_) (Figure S12, Supporting Information). Leveraging the power of spatially resolved imaging, we estimate the potential performance gain achievable by reducing *R*_S_. In an ideal scenario with negligible *R*_S_, the voltage at maximum power point condition *V*_MPP_ could reach 1.755 V, corresponding to a theoretical *PCE* of 30.9%. Realistically, if the device fabrication is optimized such that the spatial *R*_S_ distribution approaches the lower 5th percentile value (5.6 Ω cm^2^), an efficiency gain of approximately 0.2% could be achieved. While these inhomogeneities may have a limited impact on the initial device efficiency, they raise significant concerns for long‐term stability. Localized defects such as those observed can serve as degradation hotspots, compromising the reliability of tandem devices over time. These observations demonstrate how *R*_S_ imaging can provide further insights for the device characterization and loss analysis. Feature B would have been overlooked in a standard PL‐based quality control under *V*_OC_ condition and feature A would probably even have been regarded as favorable due to the decreased non‐radiative recombination, overlooking the downside of increased *R*_S_. These results illustrate the practical utility of the method for guiding design and process optimization. The ability to resolve and quantify spatial resistance variations provides actionable insight into loss mechanisms and performance ceilings – critical for advancing the commercial viability of perovskite/silicon tandem photovoltaics.

#### 3.2.2 Influence of Perovskite Hysteresis on *R*_S_ Imaging Accuracy

**FIGURE 10:**
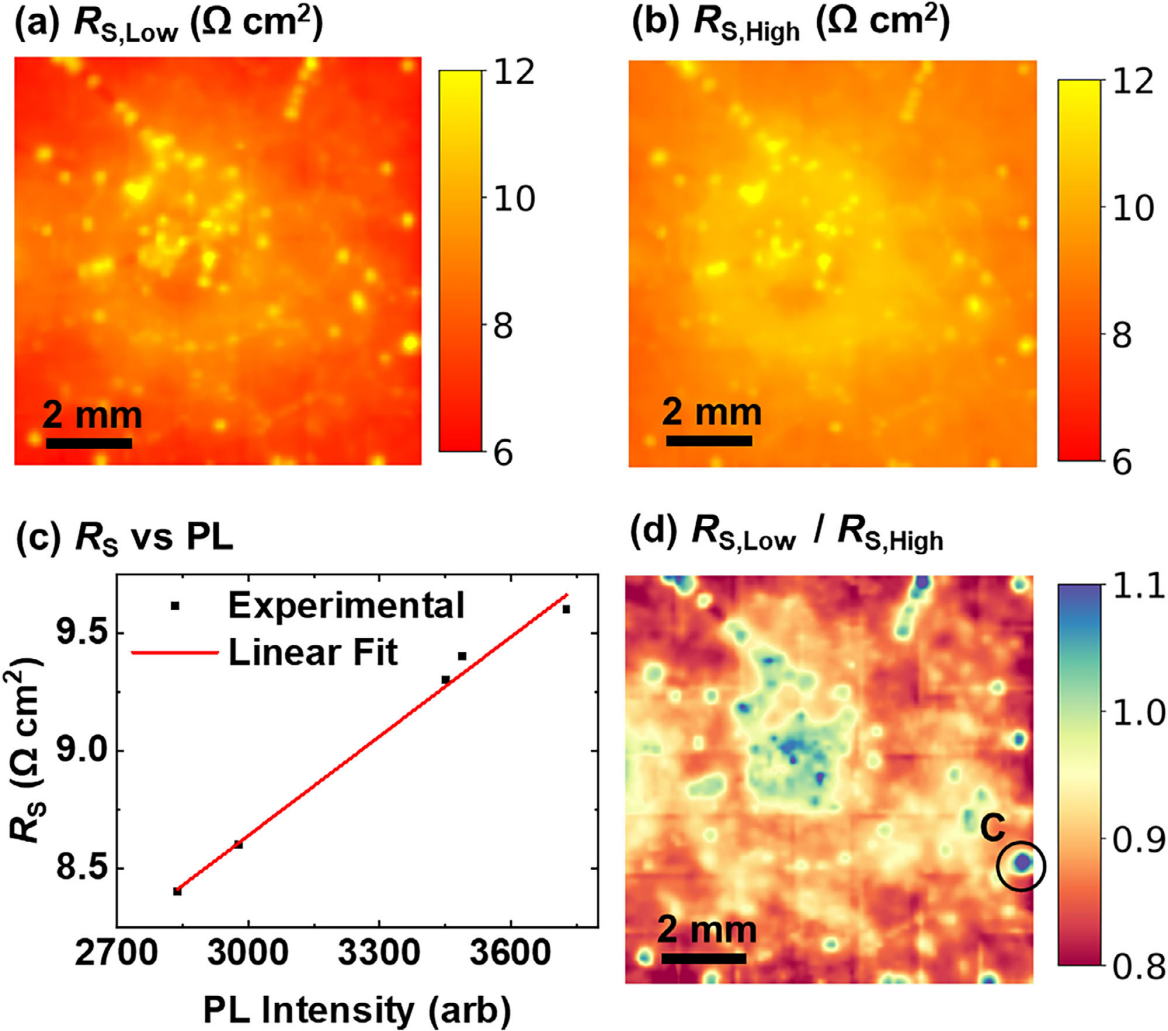
*R*_S_ images based on different PL images of the top cell measured under 1 sun and *V*_MPP_ condition but with different preconditioning, with (a) the lowest PL intensity, and (b) the highest PL intensity. (c) Correlation plot between the arithmetic mean value of *R*_S_ and PL intensities of the top cell at *V*_MPP_ of the device under 1 sun illumination, captured during stabilization. (d) Ratio image between *R*_S,Low_ versus *R*_S,High_.

A key challenge in characterizing perovskite‐based solar cells is the presence of hysteresis, primarily caused by mobile ions responding to measurement parameters such as illumination and applied bias. This transient behavior can significantly affect extracted parameters, including *V*_OC_, *FF*, and *PCE*, especially under non‐equilibrium conditions. To assess the impact of transient behavior on the accuracy of our *R*_S_ imaging method, we examined a deliberately unstable tandem device exhibiting a prolonged stabilization behavior. The EQE of a similar tandem solar cell to the device under test is displayed in Figure S11b, Supporting Information. The *jV* curve of the solar cell under standard testing conditions is displayed in Figure S13 with the *jV* parameters displayed in Table S3. To ensure the most stable condition possible during the measurement, the lasers were running continuously throughout the measurement (unless the system had to be opened to exchange optical filters for a few seconds), and the duration of bias voltage conditions, which resulted in a negative voltage for the top cell was reduced to a minimum (a few seconds). To monitor the state of the tandem solar cell and detect transient behavior, several PL images of the top cell were acquired under *V*_MPP_ condition repeatedly during the imaging procedure. Figure 10a,b displays the *R*_S_ image corresponding to the PL image with the lowest and highest PL intensities of the total series of acquired PL images acquired under approx 1 sun illumination, and *V*_MPP_ with different preconditioning histories, respectively. The PL image under *V*_MPP_ condition corresponding to *R*_S_ in Figure 10a was acquired at the beginning of the measurement procedure after some preconditioning time with continuous illumination and positive bias voltage applied beforehand. In contrast, the PL image under *V*_MPP_ condition corresponding to *R*_S_ in Figure 10b was acquired few seconds after the background image (with a terminal voltage of −0.5 V and a top cell limiting condition) was captured. Though the duration of the reverse bias condition was only about 20 s, a significant effect on the PL intensity and thus on the extracted *R*_S_ can be observed. Figure 10c shows the relationship between the arithmetic mean of *R*_S_ and the corresponding PL intensity of the top cell under 1 sun and *V*_MPP_ for all captured PL images with this same setting. A clear linear trend is observed: a ∼30 % increase in PL intensity leads to a ∼15% increase in extracted *R*_S_. In addition to the overall shift in *R*_S_ between Figure 10a,b, the spatial distribution of heterogeneity features also changes noticeably. To visualize these differences more clearly, we compute a ratio image between the *R*_S_ images in Figure 10a,b. The result is displayed in Figure 10d. In principle, if all variations were monotonic, the ratio at every pixel would be constant and remain below 1. However, we identify local regions (e.g., at Feature C in Figure 10d) where this ratio exceeds 1, confirming that different regions may even respond with a different trend to disturbances of the preconditioned state by, e.g., negative bias voltage in the perovskite subcell during the acquisition of a background image. While the root cause of Feature C can only be speculated on, a material inhomogeneity due to, e.g., agglomerates in the solution of the passivation agent could have caused this local defect. Notably, the variation for almost all pixels is in the range of less than ± 10%, indicating that with a thoroughly designed measurement procedure, at least the order of magnitude of *R*_S_ can also be identified for this unstable device. These findings highlight the importance of ensuring steady‐state conditions during the measurement. For unstable devices that require extended stabilization, it is critical to maintain continuous illumination and reduce the duration of reverse bias for the top cell to a minimum. Repeated PL measurements under the same conditions should be used to monitor the transient behavior. Without these precautions, the extracted *R*_S_ values – both in magnitude and spatial distribution – may not reflect the true steady‐state behavior of the device, compromising the method's diagnostic accuracy. Generally, the relative measurement uncertainty will be particularly high for devices with pronounced hysteresis and very low *R*_S_. While *R*_S_ of silicon single‐junction solar cells below 1 Ω cm^2^ was quantified [35] based on this PL‐based approach, the detection limit for perovskite/silicon tandem solar cells is increased due to the contribution of transient effects to the signal‐to‐noise ratio and varies for different investigated solar cells.

## 4 Conclusion and Outlook

With the LTR_S_ method, this study presents for the first time a PL‐based approach to spatially resolve the *R*_S_ in monolithic perovskite/silicon tandem solar cells. The *R*_S_ Images are extracted by capturing a series of PL images under varying bias voltages of both subcells for two different illumination intensities. The generalized Plank's law is exploited to deduce the internal voltage change from a change in PL signal intensity. The LTR_S_ method is quantitatively validated through independent finite element simulations and shown to reliably capture *R*_S_ contributions from both subcells, regardless of which subcell is current‐limiting. We further validate that LTR_S_ results are independent of the presence of shunt resistance and the luminescence coupling effect. We demonstrate the importance of acquiring an appropriate background PL image under applied bias conditions, as it plays a critical role in accurately calculating the *R*_S_ distribution.

Applying the LTR_S_ method to a perovskite/silicon tandem solar cell experimentally, the method reveals resistive features in the device that are not apparent in PL images taken under open‐circuit conditions, highlighting its diagnostic value. Furthermore, by evaluating the local *R*_S_, the potential upper limit of device performance is estimated for the case of the investigated tandem solar cell with minimized resistive losses. Finally, the influence of perovskite hysteresis on the accuracy of the method is investigated, demonstrating the need for proper stabilization protocols to ensure that the results are reliable. These findings establish the LTR_S_ method as a powerful tool for spatially resolved performance analysis and provide practical guidelines for its application to emerging tandem photovoltaics. It provides a robust and accessible framework for diagnosing performance‐limiting resistive losses and sets the stage for further developments in perovskite/silicon and other emerging photovoltaic technologies. With this, the LTR_S_ method allows for a significant advancement in the spatial analysis of series resistance in tandem devices.

## Author Contributions

Oliver Fischer and Anh Dinh Bui conceived the idea, designed the experiments, interpreted the measurement results, and wrote the manuscript. Oliver Fischer acquired the measurement data. Anh Dinh Bui evaluated the measurement data. Anh Dinh Bui and Oliver Fischer contributed equally to the manuscript. Yan Zhu, Shuai Nie, and Tanushree J.B. Nath helped acquire the first proof‐of‐concept measurements. Jann B. Landgraf, Yi Hui Hou, Wei Wang, Khoa Nguyen, Ary Anggara Wibowo, provided the investigated perovskite/silicon tandem solar cells. Juliane Borchert, Florian Schindler, Heping Shen, Klaus Weber, Hieu T. Nguyen, Stefan W. Glunz, Ziv Hameiri, Martin C. Schubert, and Daniel Macdonald provided the funding, supervised the project, and gave essential suggestions about the experimental planning, conceptualization, and structure of the manuscript. All authors revised the manuscript.

## Conflicts of Interest

The authors declare no conflicts of interest.

## Supporting information




**Supporting File**: smll72967‐sup‐0001‐SuppMat.pdf.

## Data Availability

The data that support the findings of this study are available from the corresponding author upon reasonable request.
