# Supplementary material for: Quantifying the Spatial Distribution of Series Resistance in Monolithic Perovskite/Silicon Tandem Solar Cells Using Voltage‐Dependent Photoluminescence Imaging
Source: Small. 2026 Feb 27;22(23):e13958. doi: 10.1002/smll.202513958 (PMC13100559; doi:10.1002/smll.202513958)
Supplement: Supplementary file 1 — Supporting File: smll72967‐sup‐0001‐SuppMat.pdf. [file SMLL-22-e13958-s001.pdf]

Supporting Information

Quantifying the Spatial Distribution of Series Resistance in Monolithic Perovskite/Silicon Tandem Solar Cells Using Voltage-Dependent Photoluminescence Imaging

Oliver Fischer[1,2,6,*], Anh Dinh Bui[3,6,*], Yan Zhu[4], Shuai Nie[4], Tanushree J.B. Nath[4], Yi Hui Hou[3], Wei Wang[3], Khoa Nguyen[3], Ary Anggara Wibowo[3], Jann B. Landgraf[1, 2, 5], Juliane Borchert[1,2], Florian Schindler[1], Heping Shen[3], Klaus Weber[3], Hieu T. Nguyen[3], Stefan W. Glunz[1,2], Ziv Hameiri[4], Daniel MacDonald[3,*], Martin C. Schubert[1,*]

1. Fraunhofer Institute for Solar Energy Systems ISE, Heidenhofstr. 2, 79110 Freiburg, Germany
2. Chair for Photovoltaic Energy Conversion, Department of Sustainable Systems Engineering INATECH, University of Freiburg, Emmy-Noether-Str. 2, 79110 Freiburg, Germany
3. School of Engineering, The Australian National University, Canberra, ACT, 2601 Australia
4. School of Photovoltaic and Renewable Energy Engineering, University of New South Wales, Sydney 2052, Australia
5. Cluster of Excellence *liv*MatS @ FIT – Freiburg Center for Interactive Materials and Bioinspired Technologies, University of Freiburg, Georges-Koehler-Allee 105, 79110 Freiburg, Germany
6. These authors contributed equally to this work.

1. Supplemental Items

Table S 1. Comparison of different interpolation methods on the calculation of R_S.

**Interpolation methods**	$\boldsymbol{R_{S,\mathrm{PL-based}}}$ (Ω cm^2)	$\boldsymbol{R_{S,\mathrm{Ground-truth}}}$ (Ω cm^2)
Linear	1.98	2.33
Quadratic	2.36	
Cubic	2.32	
Cubic spline	2.32	

Table S 2. Summary of the simulation parameters in **Section 3.1** in the main manuscript.

**Parameter**	**Value**							
	**Current matching w/o feature**	**Current matching w feature**	**Top cell limit (no shunt)**	**Bottom cell limit (no shunt)**	**Heavily top cell limit (no shunt)**	**Top cell limit (shunt)**	**Top cell limit (LC)**	**Bottom cell limit (LC)**
Active Area (cm^2)	100	100	100	100	100	100	100	100
Top cell wafer internal series resistance (Ω cm^2)	1	1*	1*	1*	1*	1*	1*	1*
Top cell internal shunt conductance (1/kΩ cm^2)	0	0	0	0	0	1*	0	0
Top cell finger sheet resistance (mΩ/sq)	3	3	3	3	3	3	3	3
Top cell layer sheet resistance (Ω/sq)	80	80	80	80	80	80	80	80
Top cell j_{SC} (mA/cm^2)	21.2	21.2	20.7	21.7	11.7	20.7	17.2	25.2
Top cell j_{01} (fA/cm^2)	1.00E-08	1.00E-08	1.00E-08	1.00E-08	1.00E-08	1.00E-08	1.60E-11	1.60E-11
Top cell j_{02} (fA/cm^2)	0.01	0.01	0.01	0.01	0.01	0.01	0	0
Bottom cell wafer internal series resistance (Ω cm^2)	0.3	0.3*	0.3*	0.3*	0.3*	0.3*	0.3*	0.3*

Bottom cell internal shunt conductance (1/kΩ cm²)	0	0	0	0	0	1*	0	0
Bottom cell finger sheet resistance (m Ω/sq)	3	3	3	3	3	3	3	3
Bottom cell layer sheet resistance (Ω /sq)	80	80	80	80	80	80	80	80
Bottom cell j_{SC} (mA/cm^2)	21.2	21.2	21.7	20.7	31.7	21.7	25.2	17.2
Bottom cell j_{01} (fA/cm^2)	5.00E+00	5.00E+00	5.00E+00	5.00E+00	5.00E+00	5.00E+00	6.30E+00	6.30E+00
Bottom cell j_{02} (fA/cm^2)	15	15	15	15	15	15	0	0
Interlayer sheet resistance (ohm/sq)	1.00E+05	1.00E+05	1.00E+05	1.00E+05	1.00E+05	1.00E+05	1.00E+05	1.00E+05
Photon coupling (fA/cm^2)	0	0	0	0	0	0	1.39E-11	1.39E-11
Figure in manuscript	Figure 2, Figure S 1	Figure 3, Figure 4, Figure S 2, Figure S 3	Figure 5, Figure S 4	Figure S 5	Figure S 6	Figure 7, Figure S 7	Figure 8, Figure S 8	Figure S 9

*With heterogeneity features

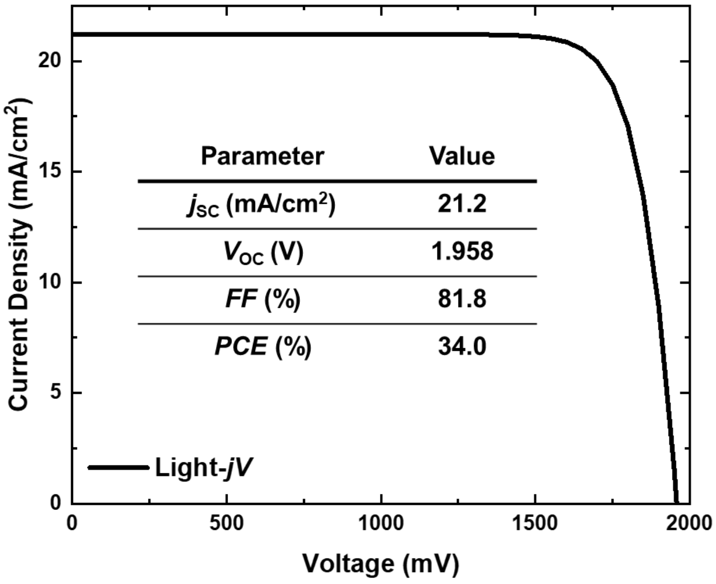

Figure S 1. Full jV curve at 1 sun and photovoltaic parameters of the simulated current-matched device, investigated in **Figure 2** in the main manuscript.

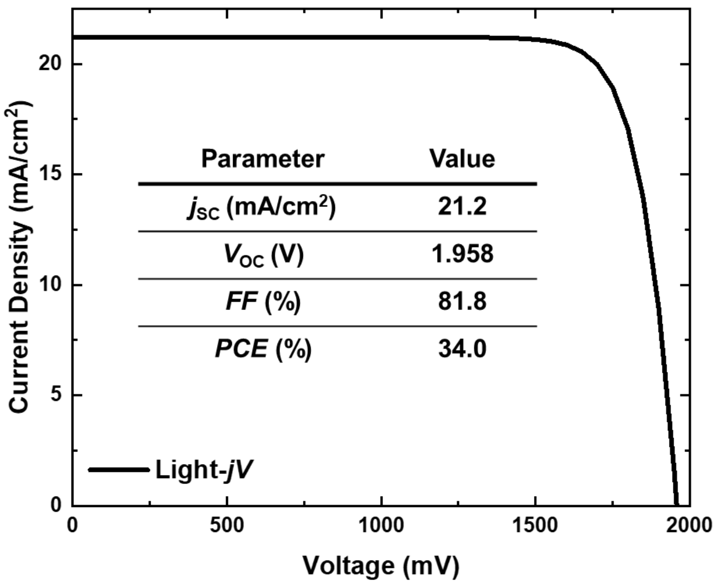

Figure S 2. Device with current-matched device with intentionally introduced resistance features, investigated in **Figure 3** and **Figure 4** in the main manuscript: full *jV* curve at 1 sun and photovoltaic parameters.

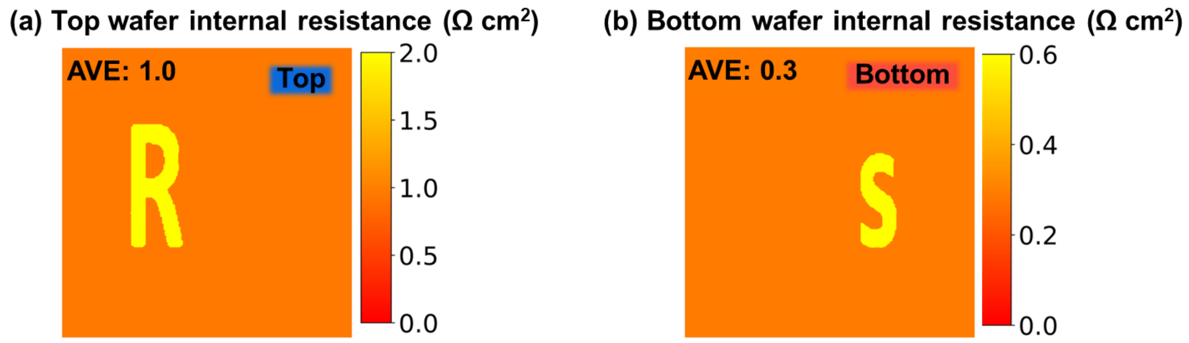

Figure S 3. Wafer series resistance of (a) top cell, (b) bottom cell. The device is simulated in current matching and intentionally introduced R_S features, investigated **Figure 3** and **Figure 4** in the main manuscript.

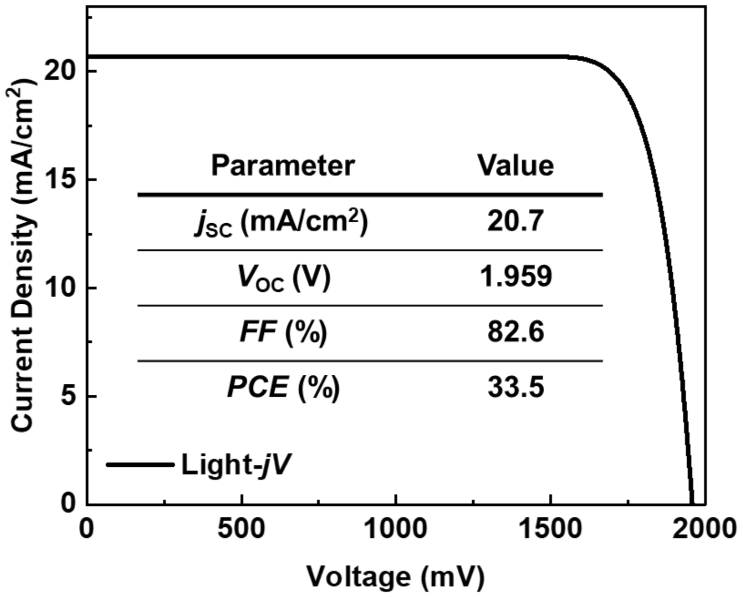

Figure S 4. Full *jV* curve at 1 sun and photovoltaic parameters of the simulated top limiting device, investigated in **Figure 5** in the main manuscript.

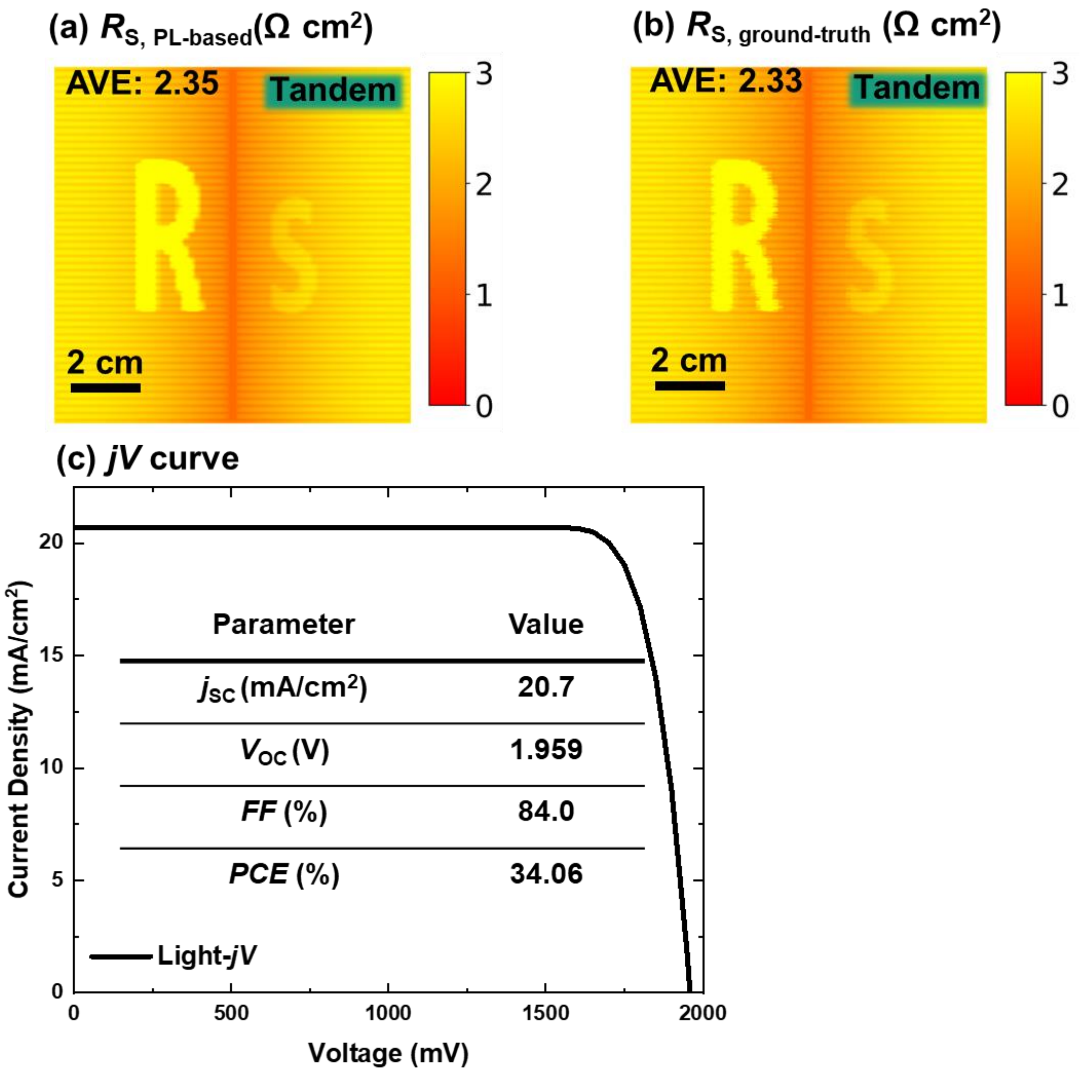

Parameter	Value
j_{SC} (mA/cm^2)	20.7
V_{OC} (V)	1.959
FF (%)	84.0
PCE (%)	34.06

Figure S 5. The device is simulated under bottom cell limiting condition and with intentionally introduced R_S features, supporting **Section 3.1.3** in the main manuscript. (a) PL-based R_S image, (b) ground-truth R_S image calculated from internal voltage images and current density images, (c) Full jV curve at 1 sun and photovoltaic parameters.

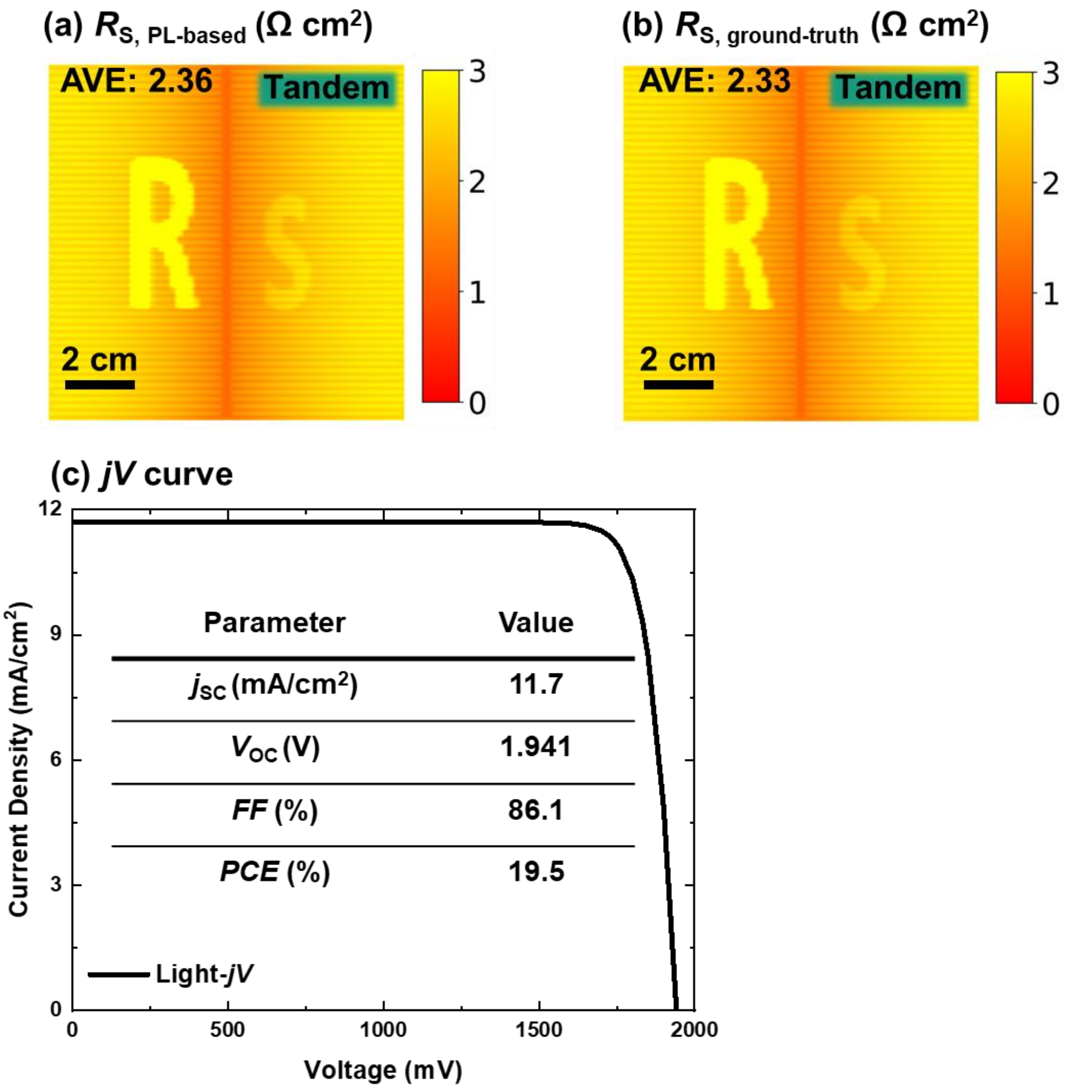

Figure S 6. The device is simulated under a heavy top cell limiting condition and with intentionally introduced R_S features, support **Section 3.1.3** in the main manuscript. (a) PL-based R_S image, (b) ground-truth R_S image calculated from internal voltage images and current density images, (c) Full jV curve at 1 sun and photovoltaic parameters.

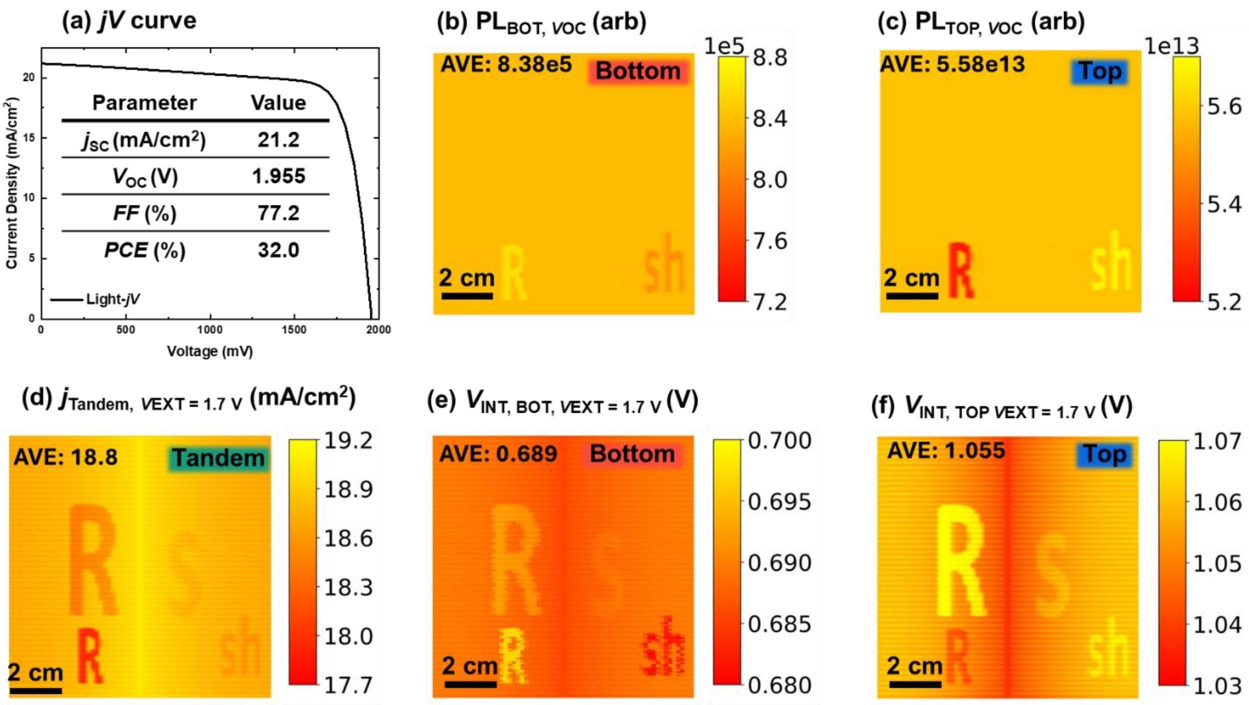

Figure S 7. Top cell limited, shunted solar cell that is investigated in **Figure 7** in the main manuscript. (a) jV curve and photovoltaic parameters at 1 sun, (b) PL image of bottom cell at V_{OC}, (c) PL image of top cell at V_{OC}, (d) j image of the device at 1.7 V, (e) $V_{\mathrm{INT,BOT}}$ image at 1.7 V, (f) $V_{\mathrm{INT,TOP}}$ image at 1.7 V.

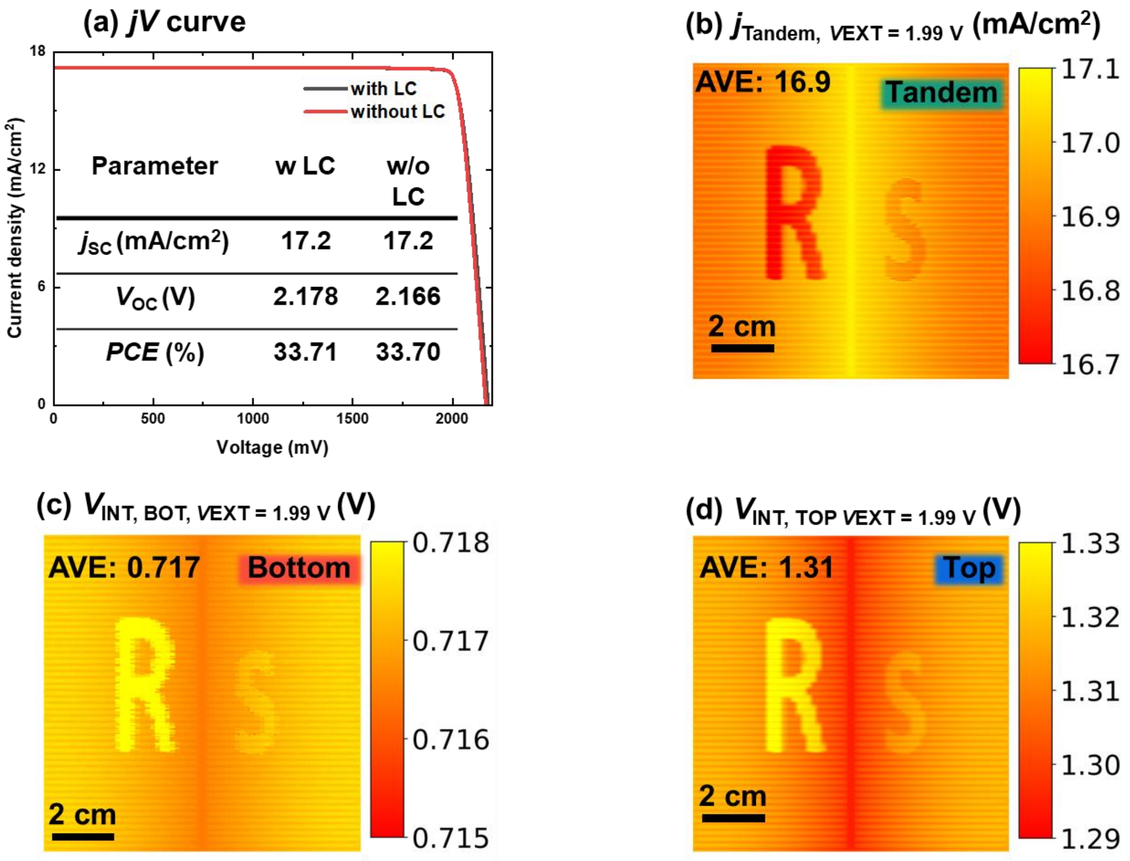

Figure S 8. Top cell limited solar cell with 87 % LC effect, investigated in **Figure 8** in the main manuscript. (a) jV curve and photovoltaic parameters at 1 sun, (b) j image of the device at 1.7 V, (c) $V_{\mathrm{INT,BOT}}$ image at 1.7 V, (d) $V_{\mathrm{INT,TOP}}$ image at 1.7 V.

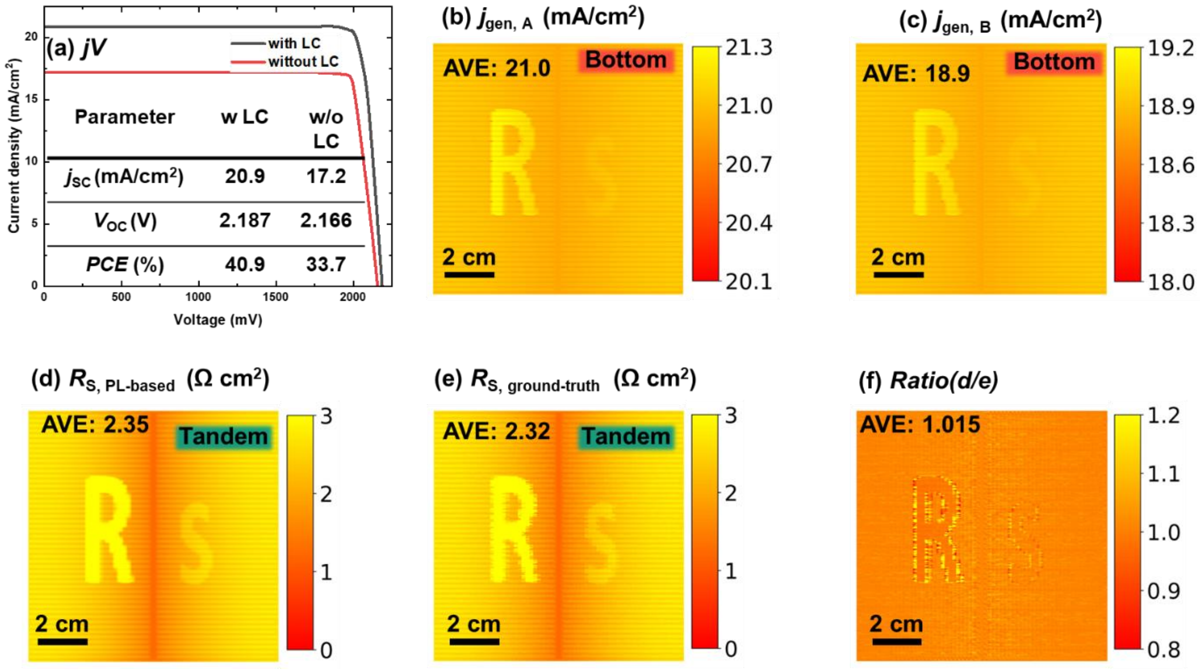

Figure S 9. Bottom cell limited solar cell with 87 % LC effect, supporting **Section 3.1.6** in the main manuscript. (a) Ratio image at V_{OC} of bottom cell of the device without and with LC effect, (b) Ratio image at V_{MPP} of bottom cell of the device without and with LC effect, (c) PL-based R_{S} image at 1.7 V, (d) ground-truth R_{S} image at 1.7 V, (e) jV curve and photovoltaic parameters at 1 sun.

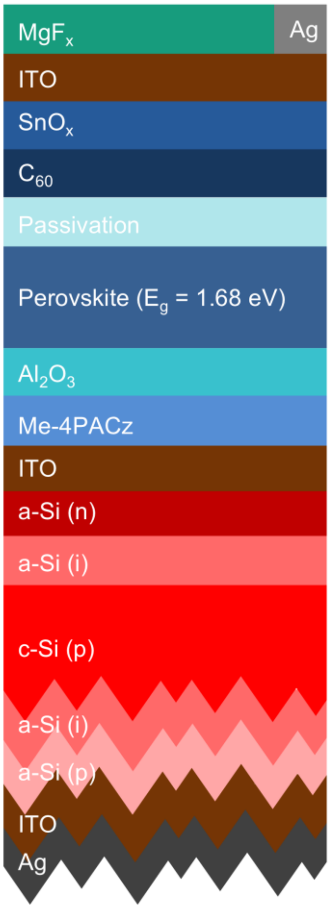

Figure S 10. Structure of the investigated perovskite/silicon tandem solar cells in **Figure 9** and **Figure 10**.

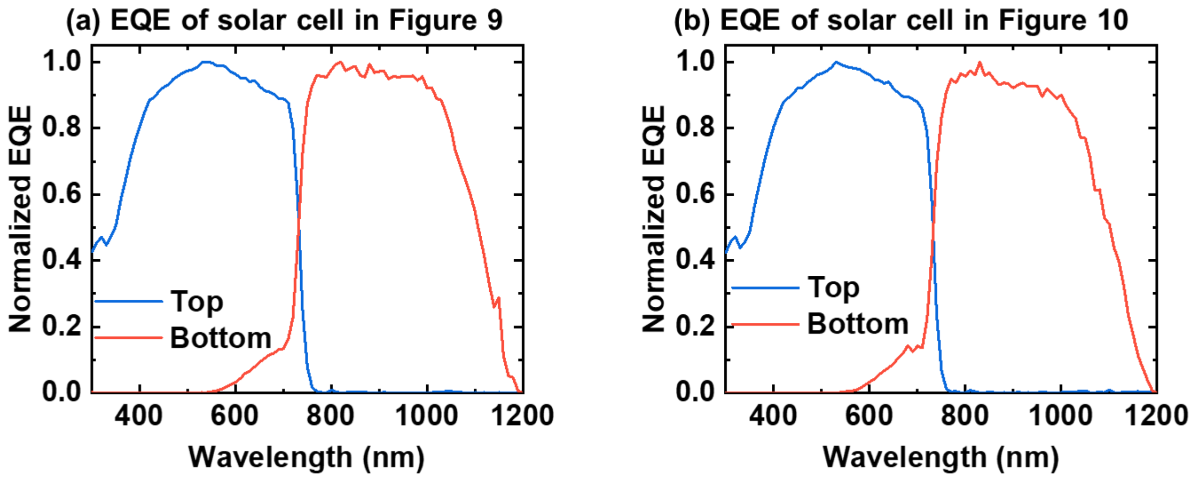

Figure S 11. Normalized EQE of a similar tandem solar cell (a) in **Figure 9** and (b) in **Figure 10.**

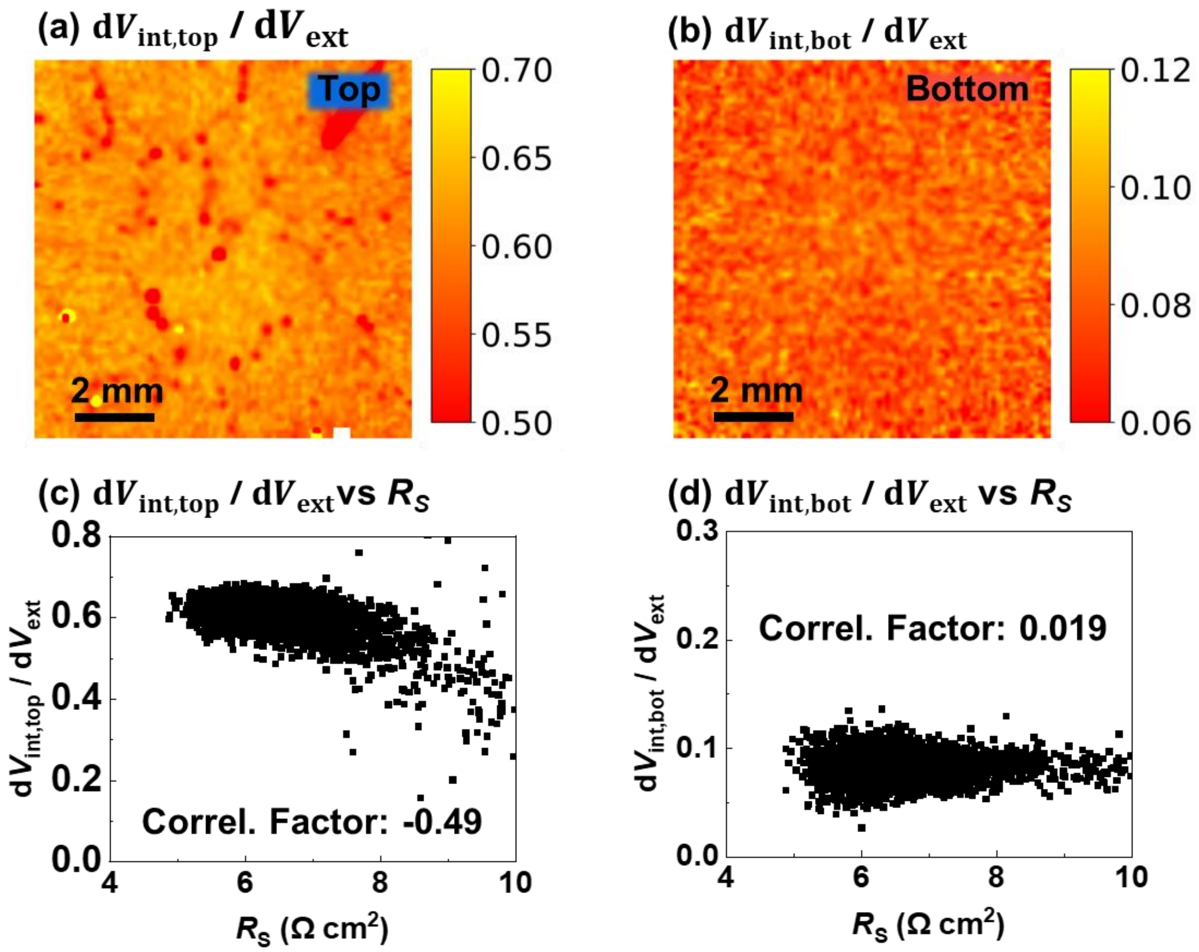

Figure S 12. (a) $dV_{int,top}$ / dV_{ext} image of the top cell, (b) $dV_{int,bot}$ / dV_{ext} image of the bottom cell, (c) correlation plot between local $dV_{int,top}$ / dV_{ext} and R_S, and (d) correlation plot between local $dV_{int,bot}$ / dV_{ext} and R_S of the device investigated in **Figure 9**. (dV_{int} / dV_{ext} $= \frac{V_t \ln(PL_{V\text{ext1}}/PL_{V\text{ext2}})}{V\text{ext1}-V\text{ext2}}$).

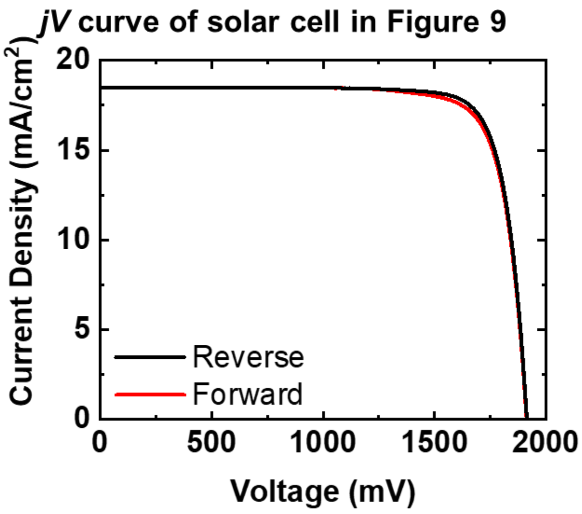

Figure S 13. jV curves of the tandem solar cell in **Figure 9** measured with an LED-based solar simulator under a spectrum adjusted to the AM1.5G spectrum.

Table S 3. jV parameters for the perovskite/silicon tandem solar cells investigated in **Figure 8** and **Figure 9** measured with an LED-based solar simulator under a spectrum adjusted to the AM1.5G spectrum.

Parameter	Stable solar cell in **Figure 8**		Transient solar cell in **Figure 9**	
	Reverse	Forward	Reverse	Forward
j_{SC} (mA/cm^2)	18.4	18.4	18.5	18.5
j_{MPP} (mA/cm^2)	17.6	17.5	17.6	17.2
V_{OC} (V)	1.914	1.912	1.914	1.913
V_{MPP} (V)	1.653	1.653	1.653	1.652
FF (%)	82.2	81.9	82.0	80.6
PCE (%)	29.0	28.9	29.0	28.5

1. Solar Cell Fabrication

Materials

Lead iodide (PbI_2), lead bromide ($PbBr_2$), and lead chloride ($PbCl_2$) were all purchased from TCI. Formamidinium iodide (FAI) was obtained from GreatCell solar, methylammonium chloride (MACl) and cesium iodide (CsI) were both obtained from Sigma-Aldrich. The [4-(3,6-dimethyl-9*H*-carbazol-9-yl)butyl]phosphonic acid (Me-4PACz) was ordered from Dyenamo, aluminum oxide nanoparticles (20 wt% in 2-propanol, particle size <50 nm) were ordered from Sigma-Aldrich, C_{60} was ordered from TCI.

Solution Preparation

The perovskite precursors for wet chemical deposition of $Cs_{0.22}FA_{0.75}MA_{0.03}Pb(I_{0.82}Br_{0.15}Cl_{0.03})_3$ perovskite with a bandgap of 1.68 eV were prepared as follows: a 1.4 M solution of $Cs_{0.22}FA_{0.78}Pb(I_{0.85}Br_{0.15})_3$ (FACs) with 5% Pb excess was made by dissolving appropriate stoichiometric amounts of FAI, CsI, $PbBr_2$, and PbI_2 in a solvent mixture of DMF:DMSO (v:v, 3:1). Moreover, a 1.4 M $MAPbCl_3$ precursor was prepared by weighing in appropriate stoichiometric amounts of MACl and $PbCl_2$ in a separate vial. The FACs solution was shaken overnight at room temperature, transferred to the vial containing $MAPbCl_3$, and heated to 60 °C for 60 min before perovskite deposition.
Solutions of Me-4PACz (1 mM, 0.33 mg mL^{-1}) were prepared by dissolving the powder in ethanol. They were left overnight at room temperature and pretreated by ultrasonication at 30 °C for 30 min prior to usage.
Aluminum oxide nanoparticles were diluted in 2-propanol to a ratio of 1:200 (v:v, Al_2O_3:IPA) and pretreated by ultrasonication at 30 °C for 60 min prior to usage.

Silicon Bottom Solar Cell

Silicon solar cells used as bottom solar cells in tandem devices were fabricated from 250 µm thick p-doped float zone silicon wafers with a base resistivity of 1 Ω cm (Siltronic). A pyramidal texture was etched on the back side of the wafer using potassium hydroxide (KOH). After RCA cleaning and subsequent dipping in an aqueous solution of 1% hydrogen fluoride (HF) to remove silicon oxide (SiO_2), an intrinsic/doped amorphous silicon passivation layer stack was deposited on both sides (plasma-enhanced chemical vapor deposition (PECVD), Indeotec cluster tool, powered at 13.56 MHz and 200 °C using mixtures of silane (SiH_4),

hydrogen (H_2), phosphine (PH_3), and trimethyl boron (TMB)). The thickness of the intrinsic layers was set to 6 nm and that of the p-doped and n-doped layers to 12 nm. Subsequently, ITO (In_2O_3/SnO_2 = 90/10 wt%) was DC sputtered on both sides using argon and oxygen mixtures (Oxford Instruments cluster tool). On the textured back side, a 195 nm ITO layer was sputtered on the full area, the recombination layer on the planar front side was formed through a 1 cm^2 shadow mask. A 1000 nm thick silver (Ag) layer was deposited on the textured side as the rear contact. Finally, the 4-inch wafers were lasered into 7 substrates with 2.5 cm × 2.5 cm dimensions, each containing one 1.1 cm × 1.1 cm ITO pad (recombination layer) to build a 1 cm^2 tandem solar cell.

Perovskite Top Solar Cell

The perovskite top solar cell has *p-i-n* polarity with a layer stack of ITO/Me-4PACz/Al_2O_3/perovskite/passivation/C_{60}/SnO_x/ITO/Ag/MgF_x. First, ethanol (200 µL) was spin coated dynamically (2500 rpm for 30 s) to clean the silicon substrate surface followed by a UV/ozone treatment for 15 min to remove contaminants. All following steps except the ITO sputtering were done under inert atmosphere. A volume of 100 µL of the Me-4PACz solution was spin coated statically (10 s waiting time, 3000 rpm for 30 s) followed by a thermal annealing treatment at 150 °C for 10 min. A SAM washing step was then incorporated by dynamically spin coating ethanol (300 µL, 4000 rpm for 50 s) to remove loosely attached Me-4PACz molecules. Next, Al_2O_3 nanoparticles (40 µL) were spin coated statically (4000 rpm for 30 s) and annealed at 100 °C for 4 min to improve the wetting for perovskite deposition. A volume of 150 µL of the perovskite solution was spin coated statically (3500 rpm for 40 s), 25 s after the start ethyl acetate (250 µL) was dropped on the substrate and the samples were annealed at 100 °C for 20 min. The perovskite layer thickness is around 550-600 nm. Next, different passivation molecules (100 µL) were spin coated dynamically (4000 rpm for 30 s) and annealed at 100 °C for 5 min. Consequently, 15 nm of C_{60} were thermally evaporated at an evaporation rate of 0.2 Å s^{-1}. A 20 nm SnO_x buffer layer was deposited at 80 °C by atomic layer deposition using deionized water (kept at room temperature) and tetrakis(dimethylamino)tin(IV) (TDMASn, kept at 50 °C) as precursors to avoid sputter damage. This was followed by the DC sputtering of 25 nm of ITO as transparent conductive oxide using a shadow mask to define an active area of 1 cm^2. Silver (300 nm) was thermally evaporated as a metal contact and subsequently MgF_x (100 nm) was thermally evaporated as an antireflection coating on the full area, both at evaporation rates of 5 Å s^{-1}.

2. Measurement Methods

PL Imaging Measurements

The PL measurements were conducted with a photoluminescence imaging (PLI) system located at Fraunhofer ISE in Germany. The commercial system was developed by Fraunhofer ISE and built by Intego GmbH. A silicon CCD camera is used for image acquisition. A 450 nm laser is used to excite the top cell and an 808 nm laser is used to excite the bottom cell. A four-quadrant power supply (Keithley 2450) from Tektronix is used for voltage biasing during the PL measurements. Optical longpass and shortpass filters are used to sharply limit the detection range of the camera. The shortpass filters (725 nm for the top cell and 1000 nm for the bottom cell) ensured that only PL signal from the high energy tail was captured, reducing the dependence of the PL signal on changes of the perovskite band gap during the measurement.
The procedure to acquire the PL images of one substrate for an $R_{S(xy)}$ image was as follows: At first, both subcells were illuminated with approximately 1 sun illumination intensity and kept at V_{OC} until the PL signal intensity and V_{OC} stabilized. Then, PL images of the top and

bottom cell were acquired under 1 sun illumination with a bias voltage of $V_{\mathrm{EXT}} = V_{\mathrm{MPP}}$ together with background PL images under 1 sun illumination and bias voltage of $V_{\mathrm{EXT}} = -0.5$ V. It was ensured that the imaged subcell was current limiting by increasing the illumination intensity for the other subcell during the background image acquisition. The duration of the application of the negative bias voltage was kept to a minimum to prevent device degradation. After each background image acquisition, the solar solar cell was kept at V_{MPP} to allow for a short regeneration. The success of the regeneration was ensured acquiring another PL image at $V_{\mathrm{EXT}} = V_{\mathrm{MPP}}$ and by tracking the extracted current. In a second step, the illumination intensity was adjusted to approximately 0.5 suns. After another stabilization period, a background image under this decreased illumination intensity with a bias voltage of $V_{\mathrm{EXT}} = -0.5$ V was acquired followed by another stabilization time again. Then, a series of PL images was acquired of the top cell starting with $V_{\mathrm{EXT}} = V_{\mathrm{MPP}} + 0.1$ V and decreasing the terminal voltage by 0.02 V for each image. In the end, a series of PL images was acquired of the bottom cell applying the same bias voltages as before. During the complete measurement procedure, the illumination was constantly switched on (interrupted by very short breaks to open the measurement system for necessary filter changes) and the bias voltage was kept in the range $V_{\mathrm{MPP}} \leq V_{\mathrm{EXT}} \leq V_{\mathrm{OC}}$ (except for few seconds during which the background images were acquired) to prevent degradation of the solar cell and reduce transient shifts during the measurement procedure.

EQE Measurements

The EQE was measured with a setup consisting of a Xenon lamp as the light source chopped at 133 Hz, a grating monochromator (to produce single-wavelength light), a transimpedance amplifier (to provide bias voltage during measurements), and a lock-in amplifier (to detect and enhance the signal/noise ratio). Prior to the measurements, a silicon reference cell was used for EQE response calibration. Then, the EQE of the tandem solar cells was measured in the range of 300 to 1200 nm (in 10 nm steps) according to Meusel et al. [1] Briefly, for measuring the silicon bottom cell, a selective blue bias light illumination was introduced together with a ~1.08 V electrical bias. For perovskite top cell measurement, a selective infrared bias light was introduced together with a ~0.68 V electrical bias.

jV Measurements

A Wavelabs SINUS 220 light-emitting diode (LED)-based solar simulator containing 20 different LEDs was used for jV measurements. The spectrum was adjusted following the procedure described by Chojniak et al.. [2] The jV curves were measured with a voltage sweep from −200 mV up to 2000 mV, using a voltage step of ~7 mV and a scan rate of 55 mV s^{-1}. All measurements were conducted in air on a temperature-controlled chuck set to 25 °C. A shadow mask was used to limit light exposure to the 1 cm^2 cell active area.

3. Suppelementary References

3.1. References

1. Meusel M, Baur C, Létay G et al. (2003) Spectral response measurements of monolithic GaInP/Ga(In)As/Ge triple-junction solar cells: Measurement artifacts and their explanation. Prog Photovolt: Res Appl 11:499–514. https://doi.org/10.1002/pip.514

2. Chojniak D, Schachtner M, Reichmuth SK et al. (2024) A precise method for the spectral adjustment of LED and multi-light source solar simulators. Prog Photovoltaics. https://doi.org/10.1002/pip.3776
